# A diethylene glycol oligomer squalene derivative modulates metabolic gene expression in ob/ob mice

**DOI:** 10.17179/excli2026-9373

**Published:** 2026-09-07

**Authors:** Yu Cheng, Munkhzul Ganbold, Elda Nurafnie Binti Ibnu Rasid, Farhana Ferdousi, Hiroko Isoda

**Affiliations:** 1 Tsukuba Life Science Innovation (T-LSI) Program, Graduate School of Science and Technology, University of Tsukuba, Tsukuba 305-8577, Japan; 2 Alliance for Research on the Mediterranean and North Africa (ARENA), University of Tsukuba, Tsukuba 305-8577, Japan; 3 Institute of Life and Environmental Sciences, University of Tsukuba, Tsukuba 305-8577, Japan; 4 Open Innovation Laboratory for Food and Medicinal Resource Engineering (FoodMed‑OIL), National Institute of Advanced Industrial Science and Technology (AIST), Tsukuba, Ibaraki 305‑0821, Japan

**Keywords:** squalene, obesity, metabolism, ob/ob mice, mitochondrial function, insulin resistance, transcriptomics, DNA microarray

## Abstract

Obesity-related metabolic dysregulation is widespread and often leads to complications such as liver dysfunction. Squalene has long been studied for its potential anti-obesity effects due to its role in lipid metabolism, oxidative stress reduction, and inflammation modulation. However, squalene's hydrophobic nature has limited its applicability in humans. Recently, an amphiphilic form of squalene, namely diethylene glycol oligomer squalene (di-SQ), has shown promise in improving metabolic functions in preadipocytes. However, the *in vivo* effects and molecular correlates of di-SQ in obesity remain insufficiently characterized. This study aimed to investigate the metabolic effects of di-SQ in an obese mouse model and to explore transcriptomic changes associated with its administration. Using the leptin-deficient ob/ob mouse model, the study employed microarray analysis, histological examination, and real-time PCR to evaluate the impact of di-SQ on obesity and associated metabolic disturbances. Di-SQ reduced body weight gain without affecting food intake and alleviated hepatic steatosis and epididymal white adipose tissue (eWAT) lipid accumulation in ob/ob mice. In the liver, di-SQ was associated with changes in lipid- and glucose-metabolism-related gene expression, energy balance, and inflammation, potentially involving hepatic *Sirt1* and *Prkaa1* expression. In eWAT, di-SQ was associated with reduced lipid accumulation, altered lipid-metabolism- and thermogenesis-related gene expression, and suppressed inflammation, thereby suggesting gene expression patterns consistent with improved insulin signaling. Di-SQ was associated with amelioration of hepatic metabolic dysfunction in ob/ob mice, potentially involving *Sirt1* and *Prkaa1*, and showed gene expression patterns consistent with enhanced insulin signaling. These findings support its potential as a novel bioactive molecule derived from natural products for obesity-related metabolic dysregulation.

See also the graphical abstract(Fig. 1).

## Abbreviations

**ATP:** adenosine triphosphate

**ALT:** alanine aminotransferase

**AST:** aspartate aminotransferase

**DAVID:** Database for Annotation, Visualization and Integrated Discovery

**DEGs:** differentially expressed genes

**di-SQ:** diethylene glycol oligomer squalene derivative

**eWAT:** epididymal adipose tissue

**GO:** Gene Ontology

**GSEA:** Gene Set Enrichment Analysis

**H&E:** Hematoxylin and eosin

**iWAT:** inguinal white adipose tissue

**LD:** lipid droplet

**NAFLD:** non-alcoholic fatty liver disease

**NCDs:** non-communicable diseases

**ORO:** Oil Red O

**PPI:** Protein-protein interaction

**RT-qPCR:** Real-time quantitative PCR

**SEM:** Standard Error of the Mean

**SQ:** Squalene

**TAC:** Transcriptome Analysis Console

**TG:** triglycerides

## 1. Introduction

As medical standards advance, numerous communicable diseases have been controlled; however, the impact of non-communicable diseases (NCDs) continues to increase. Notably, obesity-related diseases have evolved into a global health concern. This condition is a metabolic disorder characterized by obesity, insulin resistance, hyperglycemia, and dyslipidemia. These disorders increase the risk of cardiovascular diseases (Reaven, 1988[51]), non-alcoholic fatty liver disease (NAFLD) (Chalasani et al., 2012[11]), type 2 diabetes (American Diabetes Association, 2019[2]), and other health challenges. Comprehensive interventions, such as lifestyle modifications (Harrison and Day, 2007[23]; Kim et al., 2022[35]), and pharmacotherapy, are vital for effectively preventing and managing metabolic syndrome. Nonetheless, handling this syndrome often needs a polypharmacy approach to address multiple metabolic abnormalities. This complexity complicates treatment strategies and poses challenges to patient adherence. Long-term medication use introduces a range of side effects (Verhaegen and Van Gaal, 2021[64]), possibly precipitating new health complications. Therefore, the development of preventive pharmaceuticals using natural food compounds has become a focus, with the aim of reducing disease risk through early intervention.

Squalene (SQ) is a naturally occurring polyunsaturated hydrocarbon that was first discovered in shark liver oil (Hernández-Pérez et al., 1997[26]; He and Corke, 2003[24]). It has shown many benefits, including inhibiting tumor growth (Rao et al., 1998[50]), modulating inflammatory processes (Güneş, 2013[22]), improving steroid metabolism (Cárdeno et al., 2015[10]), and supporting cardiovascular health (Strandberg et al., 1990[60]). However, due to its hydrophobic structure, SQ has low bioavailability, which limits its biological function (Lohner et al., 1993[40]). To enhance the hydrophilicity of SQ, amphiphilic diethylene glycol oligomer squalene (di-SQ) was synthesized (Ngoc Linh et al., 2023[44]). As a newly synthesized compound, di-SQ has been examined only in our previous* in vitro* study (Cheng et al., 2024[12]), which demonstrated its beneficial effects on 3T3-L1 preadipocytes, including improvements in mitochondrial function, glucose metabolism, and lipid metabolism. To date, no other *in vitro *or *in vivo* studies have investigated its metabolic effects. Therefore, an early-stage exploration *in vivo* study was necessary to begin to uncover its potential physiological roles.

The ob/ob mouse is a commonly used model for studying obesity and metabolic diseases (Barton et al., 2021[5]). Due to a mutation in the leptin gene, it is unable to produce functional leptin. This defect results in hyperphagia, reduced energy expenditure, and ultimately severe obesity (Picó et al., 2022[46]). As a widely used metabolic disease model, ob/ob mice exhibit impaired mitochondrial function, characterized by reduced mitochondrial abundance and decreased oxidative phosphorylation efficiency (de Oliveira et al., 2014[15]). They also exhibit dysregulated lipid metabolism, excessive fat accumulation, and impaired lipolysis (Zechner et al., 2012[69]). Additionally, they suffer from insulin resistance and glucose metabolism disorders (Chlouverakis and White, 1969[13]), accompanied by elevated levels of pro-inflammatory factors in tissues (Rohm et al., 2022[53]). Together, mitochondrial dysfunction, lipid accumulation, insulin resistance, and chronic inflammation drive the development of complex metabolic abnormalities in ob/ob mice.

Given the metabolic functional improvements observed in preadipocytes, this exploratory study aimed to evaluate the potential of di-SQ to alleviate obesity-related metabolic dysregulation in obese mice and to explore the molecular changes associated with its administration using comprehensive gene expression profiling. As the first attempt to assess its physiological relevance, this work will provide a foundational step toward understanding the metabolic implications of di-SQ *in vivo*.

## 2. Materials and Methods

### 2.1. Chemicals

SQ was acquired by Tokyo Chemical Industry Co., Ltd. The synthesis of di-SQ followed previous protocols, as detailed in the reference (Ngoc Linh et al., 2023[44]).

Orlistat (Product No.: TRC-O686500, LGC Standards) is a widely used over-the-counter anti-obesity drug with a well-defined mechanism of action. It inhibits gastrointestinal lipases, thereby reducing fat absorption. Due to its weight-loss effects, Orlistat also contributes to the improvement of obesity-induced metabolic disorders when combined with a low-calorie diet and exercise (Nakou et al., 2010[43]; Tonstad et al., 1994[62]; Drent et al., 1995[17]; Shi et al., 2022[58]).

### 2.2. Animals and dietary regimens

The animal experiments received approval from the University of Tsukuba's Animal Study Committee (Approval No. 23-372) and were conducted in accordance with the Physiological Society of Japan's ethical guidelines for the care and use of animals in physiological research. The study is reported in accordance with ARRIVE (Animal Research: Reporting of *In Vivo* Experiments) guidelines.

**Figure 2:**
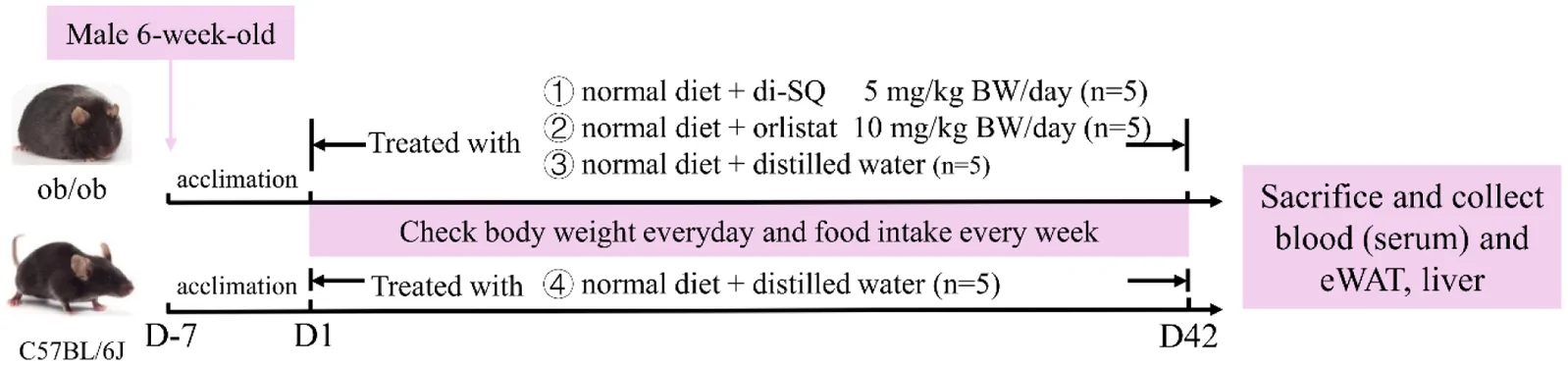
Study design: All mice were fed a normal diet and had unrestricted access to distilled water for 7 weeks. After a one-week acclimation period, the orlistat positive control group received 10 mg/kg body weight of orlistat by oral gavage, the di-SQ treatment group received 5 mg/kg body weight of di-SQ dissolved in water by oral gavage, while the ob/ob negative control group and wild-type mice which used as the baseline for the microarray experiment received an equivalent volume of distilled water by oral gavage for six weeks. At the end of the study, the mice were euthanized, and serum was collected, along with liver and eWAT samples for further analysis.

Six-week-old, male, C57BL/6J (wild type, n = 5) and C57BL/6JHamSlc-ob/ob (ob/ob, n = 15) mice (Japan SLC, Inc) were housed in approved animal care facilities (21-22 °C, constant humidity, reverse 12/12-hour light-dark cycle, with unrestricted access to water and chow food). After a one-week acclimatization period, ob/ob mice were randomly divided into three groups (n = 5 /group). Each group received daily oral gavage treatment in the morning for 42 days. Mice in the ob/ob group received distilled water, mice in the orlistat group received orlistat at 10 mg/kg body weight/day (Lee et al., 2019[38]), and mice in the di-SQ treatment group received di-SQ at 5 mg/kg body weight/day. All treatments were administered at a fixed volume of 10 mL/kg body weight. In addition, a wild-type group (n = 5) receiving distilled water was included as a baseline for the microarray experiment (Figure 2(Fig. 2)). Mice were housed by 2 to 3 animals per cage. Food intake was measured per cage and expressed as the average food intake per mouse by dividing the total food consumption by the number of mice in each cage.

Body weight in all groups was measured daily, and food intake was measured weekly. At the end of the experiment (day 42), mice were fasted for 4 hours before sample collection. A short fasting period was intentionally used to minimize metabolic alterations caused by prolonged fasting and to reduce the risk of hypoglycemia in ob/ob mice. Blood samples were collected 8 hours after the final oral administration. Animals were anesthetized with isoflurane via a nose cone, and whole blood was collected by sterile cardiac puncture. Animals were then euthanized by exsanguination under anesthesia, and organ samples were harvested. The serum was separated by centrifugation at 3000 rpm for 10 minutes and stored at -20 °C until biochemical analysis.

The liver and epididymal lchwhite adipose tissue (eWAT) were harvested, weighed, photographed, and promptly preserved in liquid nitrogen. Then, part of the liver and eWAT were frozen at -80 °C for further analysis, while the remaining tissues were fixed in FSC22 Clear Frozen Section Compound purchased by Leica Biosystems for histological studies.

### 2.3. Microarray analysis

The whole genome microarray analysis of liver tissue and eWAT collected from wild type, ob/ob, di-SQ of mice was performed on Affymetrix's GeneAtlas® System (Affymetrix Inc., Santa Clara, CA, USA), according to instructions provided by the manufacturer.

First, the total RNA was isolated from liver tissue and eWAT by ISOGEN. cDNA was synthesized by GeneChip WT PLUS Reagent Kit (Thermo Fisher Scientific, MA, USA). Subsequently, in vitro transcription, quantification, purification, and reverse transcription of cRNA were performed. Next, the synthesis, purification, fragmentation, and labeling of single-stranded cDNA were performed. Fragmented and labeled single-stranded cDNA (ss-cDNA) samples were hybridized using the cartridge array (Clariom S array, mouse; Thermo Fisher Scientific, MA, USA) on the GeneChip™ Fluidics Station and scanned by GeneChip Scanner (Thermo Fisher Scientific, MA, USA). Washing, staining, and scanning of the hybridized arrays were performed using GeneChip™ Hybridization, Wash, and Stain Kit.

For microarray analysis, liver and eWAT samples from three mice per group (n = 3) were randomly selected from each experimental group. This sample size was used for exploration transcriptomic profiling.

### 2.4. Microarray data processing and subsequent analyses

Raw image data were processed and normalized using Transcriptome Analysis Console (TAC) software (version 4.0.2, Thermo Fisher Scientific, MA, USA) with the signal space transformation robust multi-chip analysis (SST-RMA) algorithm, including background correction with GC Correction (version 4). Gene-level analysis was performed using the Limma Bioconductor package.

Microarray quality control was conducted according to standard analytical procedures. RNA samples were evaluated for quality prior to hybridization, and only samples meeting quality requirements were included. Array-level quality assessment and normalization were performed using the SST-RMA algorithm within the TAC software. All samples were processed in parallel to minimize technical variability, and no batch effects were detected or corrected. Differential gene expression analysis was performed using nominal p-values without false discovery rate correction, reflecting the exploration and hypothesis-generating design of the microarray analysis.

Differentially expressed genes (DEGs) were defined as genes with FDR < 0.05 and fold-change > 1.1, and analyzed using the functional annotation tools of Database for Annotation, Visualization and Integrated Discovery (DAVID, 2021) online bioinformatics tools (Huang et al., 2007[28]) to identify enriched Gene Ontology (GO) terms and KEGG pathways (Kanehisa et al., 2016[32]). The transcriptomic data set is available in the NCBI Gene Expression Omnibus under accession number GSE281432. Heatmaps of various pathways, generated from microarray signal intensity data, were visualized using the SRplot online tool. The Gene Set Enrichment Analysis (GSEA) plots were generated by the ExpressAnalyst online platform. Protein-protein interaction (PPI) networks were created using the NetworkAnalyst online platform. The website links and access dates are included in the attachment (Supplementary Method 1).

### 2.5. Tissue staining

Hematoxylin and eosin (H&E) stain: Sections of liver (5 µm) and adipose tissue (18 µm) were prepared from cryopreserved fresh tissue using a Leica microtome (Model DM500; Leica Microsystems, IL, USA). Tissue sections were fixed in 10 % formalin for 10 minutes, stained with hematoxylin and eosin Y (Sigma-Aldrich, Procedure No. HT110), thoroughly rinsed with running water, mounted, and observed. The area of eWAT adipocytes was quantified in ImageJ via manual measurements, and the results were validated across three independent experiments.

Oil Red O (ORO) Stain: Liver tissue sections were obtained similarly. Slides with liver sections were stained with freshly prepared Oil Red O (Sigma-Aldrich, MAK560), washed with 60 % isopropanol and mili Q water, mounted, and then observed.

### 2.6. Serum analysis / biochemical assays

Serum biochemical analyses were conducted as previously described. Glucose and triglyceride (TG) levels were measured using assay kits from Fujifilm Wako Pure Chemical Corporation. Alanine aminotransferase (ALT) and aspartate aminotransferase (AST) were analyzed using activity assay kits from Elabscience, Inc.

Hepatic TG levels were measured by the Triglyceride Assay Kit obtained from Fujifilm Wako Pure Chemical Corporation. Absorbance measurements were taken, and calculations were performed according to the manufacturer's instructions. All assays were conducted in triplicate.

### 2.7. RNA isolation and real-time quantitative PCR

Total RNA from liver tissue and eWAT were extracted using the ISOGEN reagent (Nippon Gene Co., Ltd., Toyama, Japan). First-strand cDNA was synthesized from 100 ng of total RNA by SuperScript III Reverse Transcriptase Kit (Invitrogen, Carlsbad, CA, USA). RNA and cDNA quantification was performed using the NanoDrop 2000 spectrophotometer (Thermo Scientific, Wilmington, DE, USA). Real-time quantitative PCR (RT-qPCR) for target gene expression was conducted using the 7500 Fast Real-Time PCR System (Applied Biosystems, Foster City, CA, USA), with TaqMan pre-designed primers and the TaqMan Gene Expression Master Mix (Life Technologies, Carlsbad, CA, USA). Primers *Gapdh* (Mm99999915_g1), *Cebpa* (Mm00514283_s1), *Pparg* (Mm00440940_m1), *Srebf1* (Mm00550338_m1), *Fasn* (Mm00662319_m1), *Pck1* (Mm01247058_m1), *Sirt1* (Mm01168521_m1), *Irs1* (Mm01278327_m1), *Slc2a2* (Mm00446229_m1), *Slc2a4 *(Mm00436615_m1), *Prkaa1* (Mm01296700_m1), *Cpt1a* (Mm01231183_m1), *Ppargc1a* (Mm01208835_m1), *Tfam* (Mm00447485_m1), *Ucp1*(Mm01244861_m1), *Prdm16* (Mm00712556_m1), *Tnf* (Mm00443258_m1),* Il6* (Mm00446190_m1), *Col1a1 *(Mm00801666_g1), *Tgfb1* (Mm01178820_m1), *Adipoq* (Mm04933656_m1) were obtained from Thermo Fisher Scientific. Relative mRNA levels were calculated using the 2-ΔΔCt method with *Gapdh* as the internal control.

### 2.8. Statistical analysis

Values were expressed as mean ± Standard Error of the Mean (SEM). GraphPad Prism 9.4.0 was used for data analysis and image processing. The data for each group were analyzed using the Shapiro-Wilk test (P > 0.05). Differences between the groups were analyzed using one-way ANOVA followed by Dunnett's post hoc test. P-values smaller than 0.05 were considered statistically significant.

## 3. Results

### 3.1. Di-SQ treatment reduced body and tissue weights without suppressing appetite

**Figure 3:**
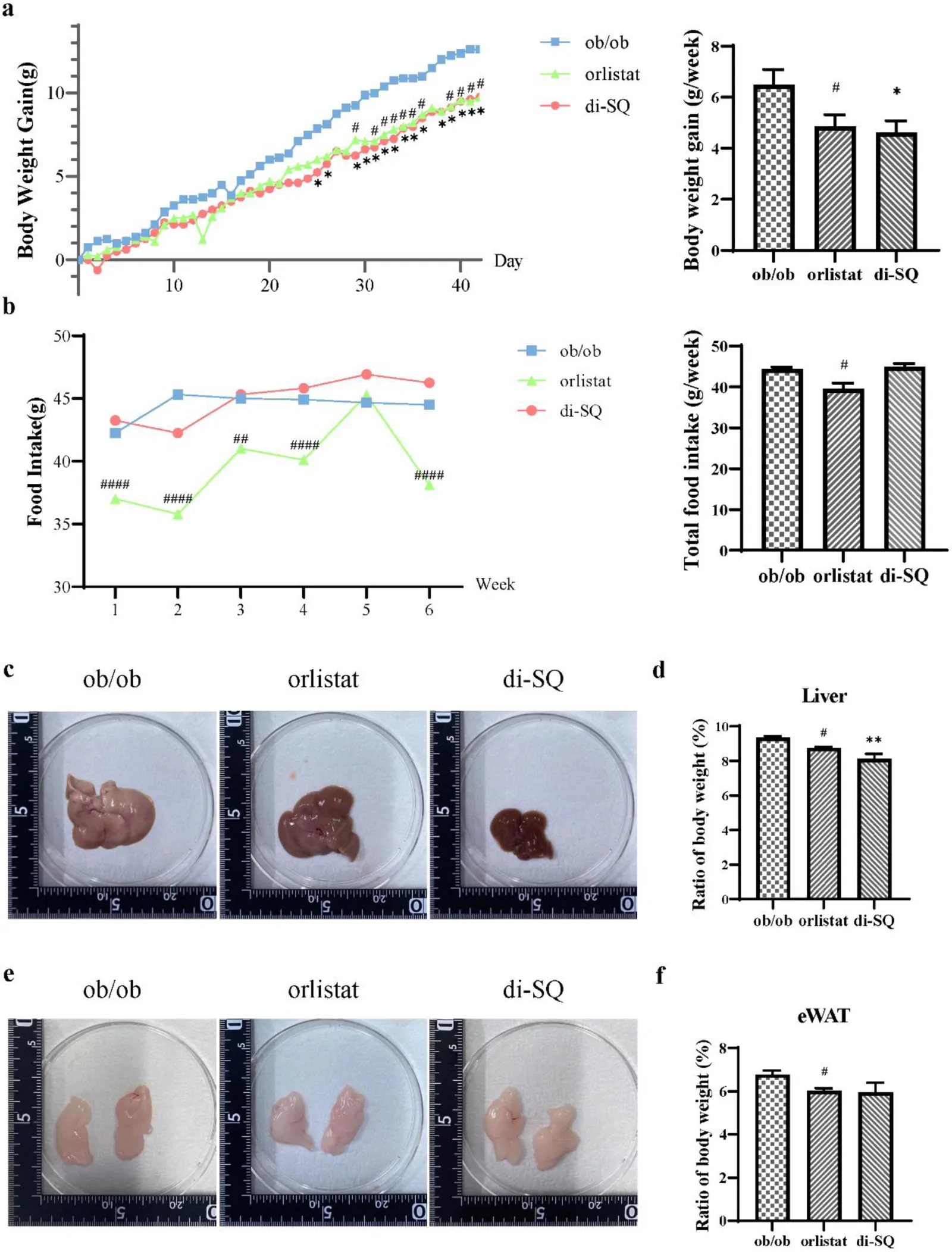
Effects of di-SQ treatment on body weight, food intake, and organ weight in ob/ob mice. (a) Body weight changes (left) and total body weight gain (right) induced by untreated ob/ob mice, orlistat treatment, and di-SQ treatment during the experimental period. Mice treated with di-SQ showed a reduction in body weight compared to ob/ob mice. (b) Food intake (left) and average food intake per week (right) induced by untreated ob/ob mice, orlistat treatment, and di-SQ treatment during the experimental period. (c) Liver morphology and (d) weight changes. Mice treated with di-SQ had visibly decreased liver size and reduced liver weight compared to untreated ob/ob mice. (e) eWAT morphology and (f) weight changes. Mice treated with di-SQ had slightly decreased eWAT size and reduced eWAT weight compared to untreated ob/ob mice (n = 5). Data were analyzed using one-way ANOVA followed by Dunnett's post hoc test. All data are presented as comparisons to the untreated ob/ob mice. Error bars represent mean ± SEM. Statistical significance: *p < 0.05; **p < 0.01 (di-SQ vs. ob/ob), #p < 0.05, ##p < 0.01, ####p < 0.0001 (orlistat vs ob/ob). n = 5 mice per group.

Over 42 days of treatment, we collected data on body weight and food intake in mice and finally harvested metabolic organs (eWAT and liver) and serum. The results showed that, compared to ob/ob mice, both orlistat and di-SQ treatments inhibited weight gain (Figure 3a(Fig. 3)). In terms of dietary intake, the orlistat treatment reduced food intake, whereas the di-SQ treatment did not alter it (Figure 3b(Fig. 3)). Consistent with the overall reduction in body weight, di-SQ treatment in ob/ob mice decreased both the liver volume and the liver-to-body weight ratio (Figure 3c, d(Fig. 3)). Similarly, eWAT showed a reduction in the eWAT-to-body weight ratio (Figure 3e, f(Fig. 3)), indicating a decrease in overall adiposity. However, compared to wild-type mice, ob/ob mice, regardless of treatment, still showed higher food intake and body weight. This observation indicated that di-SQ treatment did not fully normalize the obesity phenotype associated with leptin deficiency (Supplementary Figure 1a, b).

These findings indicate that di-SQ treatment is associated with reduced adiposity and improved liver-related parameters without suppressing food intake. However, di-SQ did not eliminate obesity-related phenotypic differences arising from genetic leptin deficiency.

### 3.2. Microarray analysis revealed that di-SQ treatment partially reversed obesity-induced lipid metabolism disorders, glucose dysregulation, and inflammation in the liver

**Figure 4:**
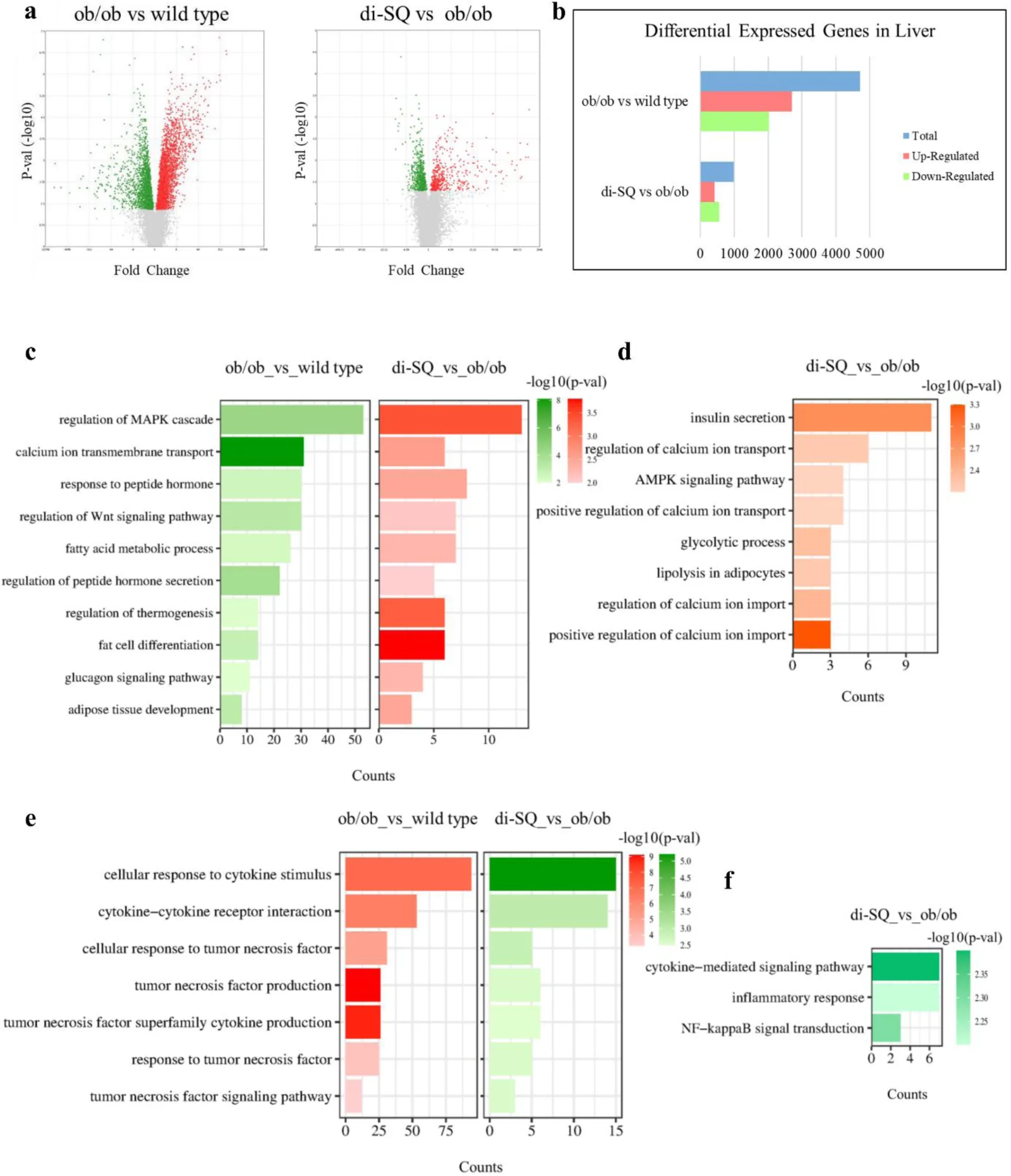
Microarray analysis of the effects of di-SQ in the liver. (a) Volcano plots for differential gene expression analysis in the liver of (left) untreated ob/ob mice compared to wild-type mice and (right) di-SQ compared to untreated ob/ob mice. Green dots indicated significantly downregulated genes and red indicated significantly upregulated genes in the di-SQ-treated group compared to the ob/ob control group. The x-axis represents the fold change in gene expression, while the y-axis represents the statistical significance (-log10 p-value). (b) Differentially expressed genes in the liver. (c) Expression levels of KEGG pathways or GO terms decreased in untreated ob/ob mice compared to wild-type mice while increased in di-SQ compared to untreated ob/ob mice. (d) Expression levels of KEGG pathways or GO terms increased by di-SQ treated which were not significantly inhibited in untreated ob/ob mice. (e) Expression levels of KEGG pathways or GO terms increased in untreated ob/ob mice compared to wild-type mice while decreased in di-SQ compared to untreated ob/ob mice. (f) Expression levels of KEGG pathways or GO terms decreased by di-SQ treated which were not significantly improved in untreated ob/ob mice. Microarray analysis was performed using liver RNA from n = 3 mice per group.

To gain a comprehensive understanding of the effects of di-SQ on liver metabolism, we conducted microarray analysis in an obese mouse model. Initial analysis revealed changes in gene expression, with 4,710 genes altered in ob/ob mice compared to wild-type mice (2,696 upregulated, 2,014 downregulated) (Figure 4a(Fig. 4)). Di-SQ treatment modified 984 differentially expressed genes (DEGs) in the liver, including 423 genes that were downregulated and 561 genes that were upregulated relative to ob/ob mice (Figure 4b(Fig. 4)), suggesting partial normalization of obesity-associated transcriptional alterations.

Functional analysis based on transcriptomic data suggested that di-SQ was associated with partial reversal of obesity-related alterations in MAPK-related pathways, thermogenesis, and fatty acid and glucose metabolism (Figure 4c(Fig. 4)). Pathways related to insulin secretion, AMPK signaling, glycolysis, and calcium ion transport showed enrichment following di-SQ treatment, whereas these pathways were not prominently enriched in ob/ob mice (Figure 4d(Fig. 4)). However, these findings should be interpreted as exploratory transcriptomic associations rather than definitive mechanistic conclusions. These results indicate transcript-level alterations consistent with metabolic pathway modulation rather than direct pathway activation. In addition to its effects on metabolism, di-SQ demonstrated potent anti-inflammatory properties. Inflammation-related pathways enriched in ob/ob mice showed reduced enrichment after di-SQ treatment (Figure 4e, f(Fig. 4)), suggesting attenuation of obesity-associated inflammatory transcriptional signatures.

Additionally, based on our previous findings regarding the effects of di-SQ on 3T3-L1 preadipocyte differentiation, di-SQ has been shown to effectively inhibit adipogenesis, reduce lipid droplet (LD) size, exhibit anti-inflammatory effects, and alleviate metabolic dysfunction in 3T3-L1 cells (Cheng et al., 2024[12]). Consistent with these observations, further analysis of microarray data showed that di-SQ treatment was associated with increased expression of genes related to mitochondrial function and insulin signaling, and reduced expression of genes associated with hepatic inflammation and lipid dysregulation (Supplementary Table 1).

### 3.3. Di-SQ treatment facilitated hepatic fatty acid balance and was associated with altered glucose metabolism related gene expression in ob/ob mice

**Figure 5:**
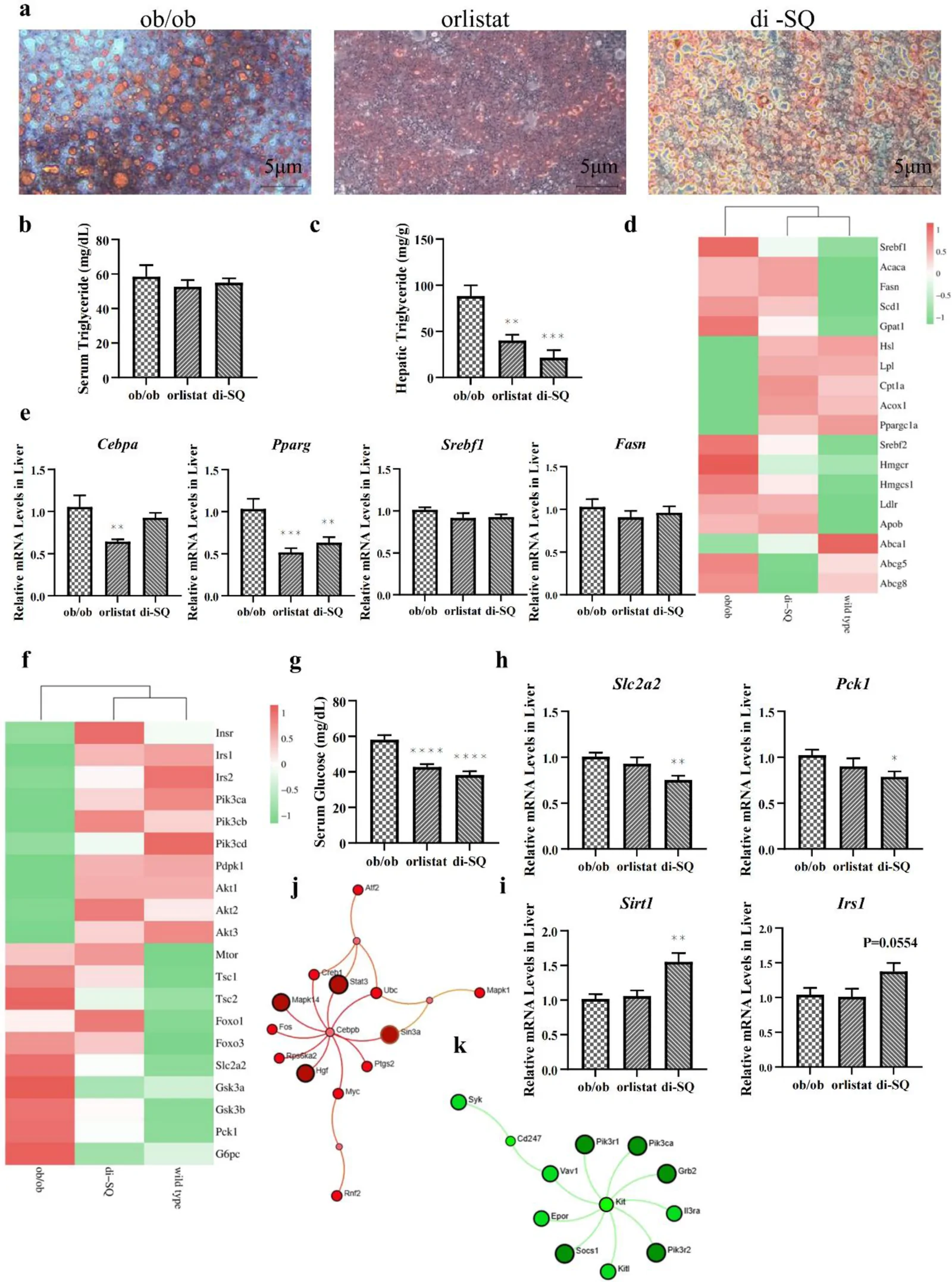
Di-SQ treatment alleviated hepatic excessive lipid accumulation and improved glucose metabolism in ob/ob mice. (locha) Oil red O stain images of liver sections from ob/ob mice, orlistat-treated mice, and di-SQ-treated mice. Scale bar = 5 μm. (b) Serum triglyceride levels and (c) hepatic triglyceride levels of each group. (d) RT-qPCR results of *Cebpa*, *Pparg*, *Srebf1*, and *Fasn* in liver treated by orlistat and di-SQ compared to ob/ob mice (n = 5). Cluster heatmaps of expression of key genes related to (e) lipid and cholesterol metabolism and (f) insulin signaling and glucose metabolism in the liver based on microarray analysis data. (g) Serum glucose levels of each group. RT-qPCR results of (h) glucose metabolism gene *Slc2a2*269 , gluconeogenesis gene *Pck1*, and (i) insulin-related genes *Sirt1* and *Irs1* in liver treated by orlistat and di-SQ compared to ob/ob mice (n = 5). Protein-protein interaction network was constructed using NetworkAnalyst, based on (j) upregulated genes from the microarray analysis, which included nodes involved in MAPK pathway, and (k) downregulated genes identified from the microarray analysis, which included proteins involved in type 2 diabetes signaling pathway and insulin resistance signaling pathway, as identified through KEGG pathway database. Data were analyzed using one-way ANOVA followed by Dunnett's post hoc test. Error bars represent mean ± SEM. Statistical significance: **p < 0.01; ***p < 0.001; ****p < 0.0001.n = 5 mice per group.

To validate and further support our microarray screening results, we initiated assessments of biochemical markers. ORO staining of liver tissue revealed a marked reduction in lipid accumulation after di-SQ treatment, especially compared to the untreated group (Figure 5a(Fig. 5), Supplementary Figure 2a-c). Although serum TG levels did not change greatly (Figure 5b(Fig. 5)), hepatic TG content decreased (Figure 5c(Fig. 5)), indicating reduced hepatic lipid accumulation following di-SQ treatment.

Next, we analyzed metabolism-related genes using microarray data and confirmed gene expression changes with a clustered heatmap. The di-SQ treatment did not reverse excessive lipid production in obese mice, particularly genes such as* Fasn* and *Acaca*. However, it reversed fatty acid mobilization and oxidation processes. Di-SQ was also associated with partial reversal of cholesterol metabolism-related gene expression, including genes involved in cholesterol synthesis and uptake (Figure 5e(Fig. 5)). Furthermore, genes involved in lipogenesis (*Cebpa*, *Pparg*, *Srebf1*, and* Fasn*) were downregulated after di-SQ treatment, with *Pparg* showing downregulation (Figure 5d(Fig. 5)), suggesting transcriptional modulation of hepatic lipogenic pathways. However, the effects on* Srebf1* and* Fasn* were less pronounced, indicating a limited or delayed response. Given the changes in fatty acid metabolism, we subsequently investigated whether di-SQ treatment also affected hepatic glucose metabolism.

Similarly, we conducted an initial screening of the signal intensities of genes related to glucose metabolism and generated a clustered heatmap. The results showed that, in most processes, mice treated with di-SQ exhibited greater similarity to wild-type mice than to ob/ob mice, while most gene expression patterns in the di-SQ group were distinctly opposite to those in the ob/ob group. Notably, genes associated with insulin-responsive pathways and gluconeogenesis displayed opposite expression trends in the di-SQ and ob/ob groups, although mTOR and some FOXO family genes did not exhibit a normalization trend (Figure 5f(Fig. 5)). These results indicated partial normalization of hepatic glucose metabolism-related gene expression patterns rather than functional pathway activation.

Serum glucose levels were measured, showing a reduction after both orlistat and di-SQ treatment, with di-SQ having a more pronounced effect (Figure 5g(Fig. 5)). RT-qPCR validation revealed that di-SQ downregulated glucose metabolism and gluconeogenesis genes* Slc2a2* and* Pck1* (Figure 5h(Fig. 5)), and upregulated insulin signaling genes *Sirt1* and *Irs1* (Figure 5i(Fig. 5)). DEG analysis using NetworkAnalyst identified pathways including MAPK- and insulin-resistance-related pathways among differentially expressed genes. These analyses highlighted transcript-level pathway associations rather than direct functional modulation (Figure 5j, k(Fig. 5)). These analyses highlighted transcript-level pathway associations rather than direct functional modulation (Hüttemann et al., 2007[29]).

In summary, di-SQ treatment partially inhibited excessive lipid accumulation and effectively suppressed the expression of cholesterol synthesis- and uptake-related genes in the liver. Alterations in hepatic glucose metabolism-related gene expression were also observed, alongside changes in fatty acid mobilization and oxidation-associated genes. These transcriptomic findings suggest coordinated regulation of hepatic metabolic pathways, prompting further investigation into mitochondrial metabolism.

### 3.4. Di-SQ treatment was associated with restoration of mitochondrial gene expression and attenuation of liver inflammation

**Figure 6:**
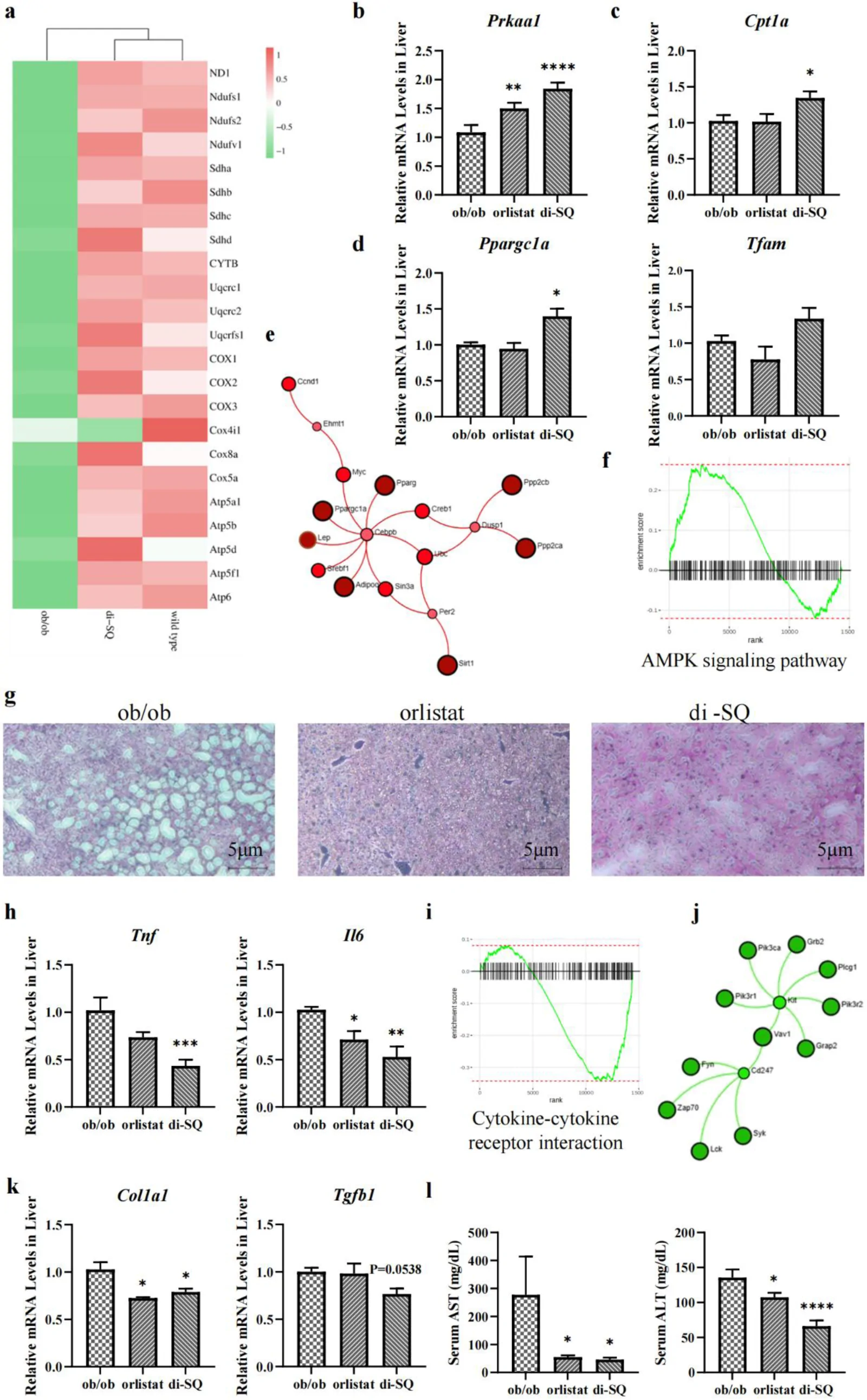
Di-SQ treatment improved hepatic mitochondrial function potentially through the AMPK pathway. (a) Cluster heatmap of expression of key genes related to mitochondrial respiratory chain in the liver based on microarray analysis data. RT-qPCR results of key genes in energy metabolism (b) *Prkaa1*, (bc) *Cpt1a*, (d) *Ppargc1a* and *Tfam *in liver treated by orlistat and di-SQ compared to ob/ob mice (n = 5). (e) Protein-protein interaction network constructed using NetworkAnalyst based on upregulated genes from the microarray analysis, which included nodes involved in the AMPK signaling pathway, as identified through the KEGG pathway database. (f) The GSEA plot showed the enrichment of the AMPK signaling pathway in the ranked gene list derived from microarray data. (g) H&E stain images of liver sections from ob/ob mice, orlistat-treated mice and di-SQ-treated mice. Scale bar = 5 μm. RT-qPCR results of (h) cytokines * Tnf* and *Il6*, and (k) fibrosis-related genes *Col1a1* and *Tgfb1* in liver treated by orlistat and di-SQ compared to ob/ob mice (n = 5). (i) The GSEA plot showed the enrichment of the Cytokine-cytokine receptor interaction in the ranked gene list derived from microarray data. (j) Protein-protein interaction network of nodes involved in TNF and NF-kappa B signaling pathway, as identified through the KEGG pathway database. (l) Liver injury biomarkers: serum ALT and AST levels of each group. Data were analyzed using one-way ANOVA followed by Dunnett's post hoc test. Error bars represent mean ± SEM. Statistical significance: *p < 0.05; **p < 0.01; ***p < 0.001, ****p < 0.0001.n = 5 mice per group.

Based on the effects of di-SQ on lipid and glucose metabolism, we became interested in how it affected mitochondrial energy metabolism in the liver. Similarly, heatmaps were generated using the signal intensities selected from the mitochondrial dataset of respiratory chain genes from microarray experiments. In the five respiratory chains, di-SQ normalized the majority of genes, except *Cox4l1* (Figure 6a(Fig. 6)). This finding suggested partial restoration of mitochondrial gene expression profiles, with *Cox4l1*, a regulatory subunit (Pajuelo Reguera et al., 2020[45]), possibly responding differently from other respiratory-chain-related genes. As a key energy-sensing enzyme in the liver, AMPK was upregulated in the pathway analysis, and *Prkaa1* (Figure 6b(Fig. 6)) was also confirmed to be upregulated in the PCR validation. Similarly, *Cpt1a*, which regulates the transport of long-chain fatty acids into mitochondria for beta-oxidation (Liang, 2023[39]), was upregulated (Figure 6c(Fig. 6)). These results indicate transcriptional changes consistent with enhanced mitochondrial fatty acid utilization. Additionally, we focused on genes related to energy metabolism and mitochondrial function in the liver. The expression of *Ppargc1a *and *Tfam*, which is strongly associated with mitochondrial function and biosynthesis, increased after di-SQ treatment (Figure 6d(Fig. 6)), suggesting modulation of mitochondrial regulatory programs at the gene expression level.

Following the approach described earlier, we constructed additional PPI networks of the AMPK pathway from upregulated DEGs (Figure 6e(Fig. 6)), and this finding was corroborated by the AMPK signaling pathway in the GSEA plot, which was generated using the ExpressAnalyst software for statistical and functional analysis of all DEGs in the livers of di-SQ-treated mice (Figure 6f(Fig. 6)). Together, these analyses indicated coordinated transcriptomic changes related to mitochondrial energy metabolism rather than direct functional activation. Since di-SQ treatment showed transcriptomic patterns consistent with AMPK pathway modulation and reduced hepatic lipid accumulation, we subsequently examined its efficacy in ameliorating liver inflammation and injury. In the H&E staining of liver tissue, di-SQ-treated liver displayed fewer cracks and reduced steatosis, as observed directly, compared to untreated ob/ob mice. (Figure 6g(Fig. 6)), whereas mice treated with orlistat exhibited improvements only in steatosis. These histological observations suggested improved hepatic morphology. To validate this conclusion, we confirmed the expression levels of the inflammatory factors *Tnf* and *Il6* (Figure 6h(Fig. 6)), the expression of both was suppressed by di-SQ treatment. Similarly, this conclusion was supported by the GSEA and PPI analysis. The GSEA plot showed the enrichment of the cytokine-cytokine receptor interaction pathway in the ranked gene list derived from microarray data (Figure 6i(Fig. 6)), indicating that the inflammatory response was suppressed and PPI analysis identified downregulation of nodes associated with TNF and NF-kappa B signaling (Figure 6j(Fig. 6)), further confirming the role of di-SQ in suppressing hepatic inflammation.

Additionally, fibrosis genes *Col1a1* and *Tgfb1 *were also downregulated by di-SQ treatment with or without significance (Figure 6k(Fig. 6)). This may be an additional result of inhibiting excessive lipid production. Furthermore, serum AST and ALT levels were markedly suppressed (Figure 6l(Fig. 6)), supporting an association between di-SQ treatment and attenuation of hepatic injury and inflammation.

### 3.5. Di-SQ treatment was associated with altered adipose gene expression patterns related to metabolism and inflammation

**Figure 7:**
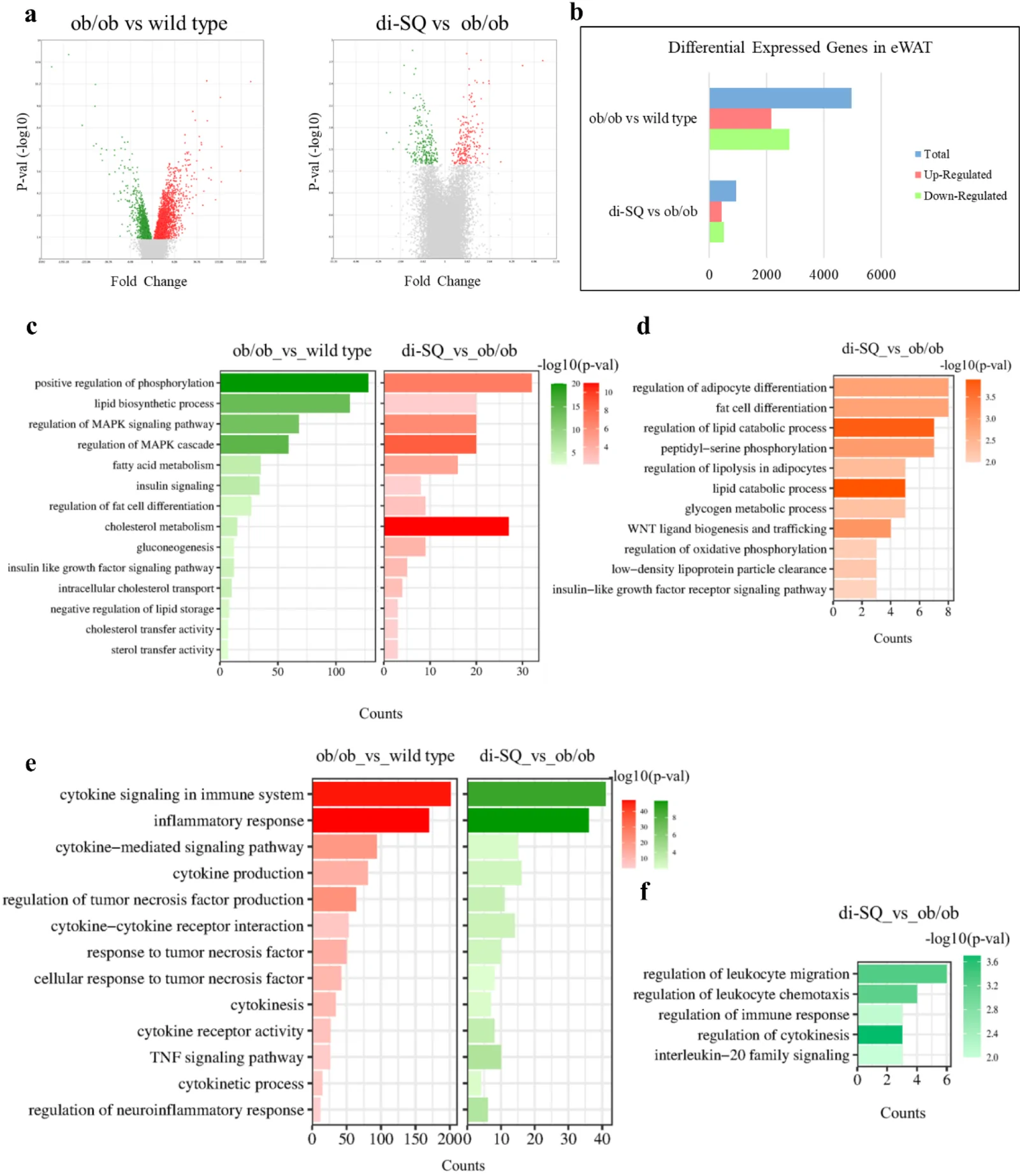
Microarray analysis of di-SQ effects in eWAT. (a) Volcano plot for differential gene expression analysis in eWAT of (left) untreated ob/ob mice compared to wild-type mice and (right) di-SQ compared to untreated ob/ob mice. Green dots indicated significantly downregulated genes and red indicated significantly upregulated genes in the di-SQ-treated group compared to the ob/ob control group. The x-axis represents the fold change in gene expression, while the y-axis represents the statistical significance (-log10 p-value). (b) Differential expressed genes in eWAT. (c) Expression levels of KEGG pathways or GO terms decreased in untreated ob/ob mice compared to wild-type mice while increased in di-SQ compared to untreated ob/ob mice. (d) Expression levels of KEGG pathways or GO terms increased by di-SQ treated which were not significantly inhibited in untreated ob/ob mice. (e) Expression levels of KEGG pathways or GO terms increased in untreated ob/ob mice compared to wild-type mice while decreased in di-SQ compared to untreated ob/ob mice. (f) Expression levels of KEGG pathways or GO terms decreased by di-SQ treated which were not significantly improved in untreated ob/ob mice. Microarray analysis was performed using eWAT RNA from n = 3 mice per group.

For eWAT, we similarly first conducted a microarray experiment to examine the overall gene expression profile. Di-SQ treatment altered the gene expression pattern in the eWAT of ob/ob mice (Figure 7a(Fig. 7)), although this change was less pronounced than in the liver. Compared to healthy wild-type mice, the expression of 4,949 genes was altered in obese ob/ob mice eWAT, with 2,158 genes upregulated and 2,791 genes downregulated (Figure 4a(Fig. 4)). These changes underscore the profound metabolic dysregulation in ob/ob mice. Di-SQ treatment, however, altered the expression of 930 DEGs in eWAT compared with the untreated ob/ob group. Among these, di-SQ restored the expression of 419 genes that were downregulated in ob/ob mice and normalized 511 genes that were upregulated in ob/ob mice (Figure 7b(Fig. 7)). Functional enrichment analysis identified exploratory transcriptomic changes in pathways related to fatty acid metabolism, gluconeogenesis, cholesterol metabolism, insulin signaling, insulin-like growth factor signaling, and MAPK-related signaling following di-SQ treatment (Figure 7c(Fig. 7)). Additionally, pathways related to adipocyte differentiation, lipid catabolic processes, lipolysis in adipocytes, glycogen metabolic processes, low-density lipoprotein particle clearance, insulin-like growth factor receptor signaling, insulin secretion, and oxidative phosphorylation showed enrichment following di-SQ treatment (Figure 7d(Fig. 7)). These findings reflect transcript-level changes in pathway-related genes rather than direct evidence of pathway activation. In addition to metabolic pathways, di-SQ treatment was associated with reduced enrichment of inflammation-related pathways that were either elevated in ob/ob mice (Figure 7e(Fig. 7)) or not prominently observed (Figure 7f(Fig. 7)).

To evaluate whether the other observed effects could be reproduced *in vivo*, we conducted a preliminary analysis of the microarray data, focusing on the normalized expression values of genes associated with adipogenesis, and metabolic disorders based on their biological significance. The results revealed that, in eWAT, di-SQ treatment was associated with altered expression of genes related to adipogenesis, mitochondrial function, insulin signaling, and diabetes-related pathways, as well as downregulated genes associated with inflammation and lipid droplet size (Supplementary Table 2).

**Figure 8:**
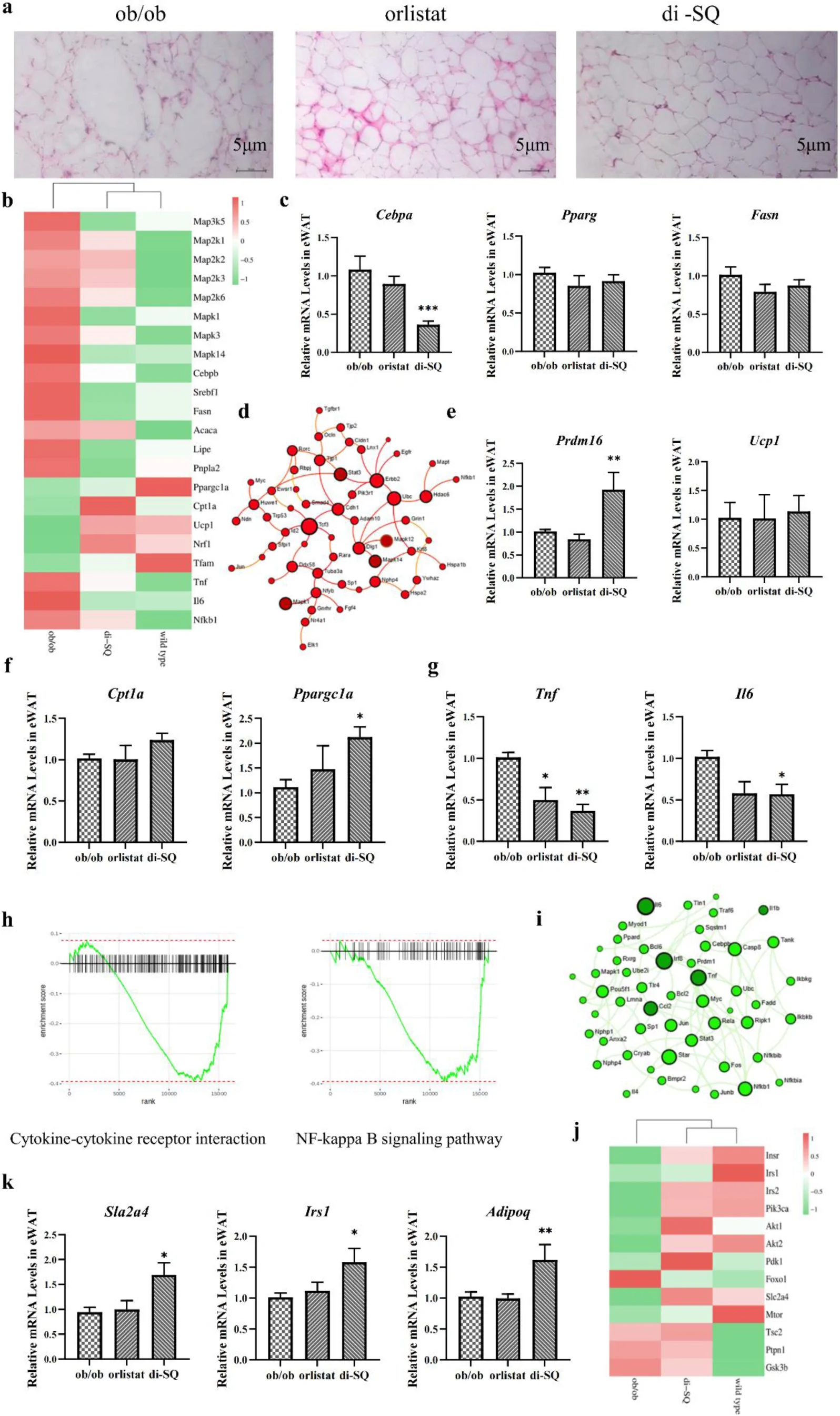
Di-SQ treatment improved the overall metabolic condition of eWAT. (a) H&E stain images of eWAT sections from ob/ob mice, orlistat-treated mice, and di-SQ-treated mice. Scale bar = 5 μm. (b) Cluster heatmaps of expression of core molecules of the ERK pathway and p38 pathway and followed energy metabolism, and inflammation. (c) RT-qPCR results of *Cebpa*, *Pparg* and *Fasn* in eWAT treated by orlistat and di-SQ compared to ob/ob mice. (d) Protein-protein interaction network was constructed using NetworkAnalyst based on upregulated genes from the microarray analysis, which included nodes involved in the MAPK signaling pathway, as identified through the KEGG pathway database. RT-qPCR results of (e) thermogenesis-related genes *Prdm16* and* Ucp1, *(f) mitochondrial function-related genes *Cpt1a* and* Ppargc1a, *and (g) inflammation-related genes *Tnf* and* Il6 *in eWAT treated by orlistat and di-SQ compared to ob/ob mice. (h) GSEA plots showed the enrichment of the Cytokine-cytokine receptor interaction and NF-kappa B signaling pathway in the ranked gene list derived from microarray data. (i) Protein-protein interaction network of nodes involved in the TNF signaling pathway, as identified through the KEGG pathway database. (j) Cluster heatmaps of expression of key genes related to insulin signaling and glucose metabolism in the liver based on microarray analysis data. (k) RT-qPCR results of glucose metabolism-related genes *Slc2a4, Irs1* and* Adipoq *in eWAT treated by orlistat and di-SQ compared to ob/ob mice. Data were analyzed using one-way ANOVA followed by Dunnett's post hoc test. Error bars represent mean ± SEM. Statistical significance: *p < 0.05; **p < 0.01; ***p < 0.001. n = 5 mice per group.

Based on preliminary microarray results, we used the MAPK pathway as a link to further examine changes in biomarkers of lipid metabolism, energy metabolism, and inflammation in eWAT to confirm the effects of di-SQ treatment. The smaller, more uniform cell size observed in the H&E staining results further indicated reduced adipocyte expansion and improved adipose tissue structure (Figure 8a(Fig. 8), Supplementary Figure 3). Given the prominent modulation of MAPK-related pathways observed in the microarray analysis (Figure 7c(Fig. 7)), we analyzed core molecules of the p38 and ERK pathways to evaluate their effects on metabolism and inflammation in eWAT (Figure 8b(Fig. 8)). Di-SQ treatment was associated with reduced expression of MAPK-related inflammatory and lipid accumulation markers, alongside increased expression of genes related to energy metabolism. As markers of adipogenesis and lipogenesis,* Cebpa*, *Pparg* and *Fasn* were examined. *Cebpa* was downregulated in the di-SQ treatment group, while *Pparg* and *Fasn* showed a slight decrease, however without significance (Figure 8c(Fig. 8)), indicating transcriptional suppression of adipogenic programs rather than complete inhibition. Similarly, upregulated DEGs from eWAT were enriched in the MAPK pathway (Figure 8d(Fig. 8)), following the same approach as described above. Genes related to thermogenesis and browning, including *Prdm16* and *Ucp1* (Figure 8e(Fig. 8)), were upregulated in eWAT. Similarly, genes related to energy metabolism, including *Cpt1a* and *Ppargc1a* (Figure 8f(Fig. 8)), were also upregulated. These results indicated coordinated changes in gene expression related to energy metabolism and thermogenic programs, although direct activation of MAPK signaling cannot be inferred from these data alone.

Next, the expression of inflammatory factors shown in the heatmap to be suppressed by di-SQ treatment was validated. The results showed that* Il6 *and *Tnf *were downregulated by di-SQ, with an effect comparable to orlistat treatment (Figure 8g(Fig. 8)). Additionally, the GSEA of the ranked gene list from microarray data revealed negative enrichment for the cytokine-cytokine receptor interaction and NF-kB signaling pathways (Figure 8h(Fig. 8)). Furthermore, a PPI network constructed from downregulated DEGs focused on the TNF signaling pathway further supported the inhibitory effect of di-SQ on inflammation-related pathways (Figure 8i(Fig. 8)).

To further validate the role of di-SQ in improving metabolism in eWAT, we next examined key genes related to the insulin signaling pathway. Di-SQ treatment was associated with partial normalization of gene expression patterns related to insulin signaling and glucose metabolism, whereas genes involved in negative regulatory processes showed limited changes (Figure 8j(Fig. 8)). Additionally, *Slc2a4*, *Irs1*, and *Adipoq*, genes related to insulin sensitivity, glucose metabolism, and adipokine secretion, were upregulated by di-SQ treatment (Figure 8k(Fig. 8)), consistent with transcriptomic patterns suggestive of improved adipose metabolic regulation. However, these findings reflect changes in gene expression levels and do not directly demonstrate functional improvements in insulin sensitivity.

## 4. Discussion

In this study, we conducted comprehensive biochemical and whole-transcriptomic analyses of di-SQ, an ethylene glycol oligomer of squalene, in an obesity mouse model, and for the first time found that di-SQ reduced body weight gain and was associated with improvements in obesity-related metabolic alterations in major metabolic organs, particularly the liver.

Obesity-related dysmetabolism can lead to non-alcoholic fatty liver disease, liver inflammation, fibrosis, and dysfunctional adipocyte activity (Stefan et al., 2025[59]). Although natural compounds such as squalene have shown potential *in vitro*, their therapeutic efficacy *in vivo* remains to be fully elucidated. In this study, we demonstrated that oral administration of di-SQ was associated with reduced body weight gain and anti-inflammatory effects. Notably, transcriptomic and gene expression analyses identified potential changes in genes related to mitochondrial function and insulin signaling. In parallel, di-SQ reduced obesity-related pathological changes in the liver and eWAT of ob/ob mice. Compared with untreated or orlistat-treated ob/ob mice, di-SQ exhibited more evident therapeutic potential in the liver, whereas its effects in eWAT were comparatively weaker.

Orlistat typically does not directly suppress appetite but promotes weight loss mainly by inhibiting gastrointestinal lipases and reducing fat absorption. However, some studies have suggested that orlistat may alter the gut microbiota (Ke et al., 2020[34]), or indirectly affect appetite through changes in gut barrier function (Katimbwa et al., 2022[33]), which may explain the reduced appetite observed in our orlistat-treated mice. Lipid droplets (LDs) in the liver are involved in lipid storage and metabolism, providing energy or triglycerides for transport to other tissues, including WAT (Heeren and Scheja, 2021[25]). In our study, di-SQ was associated with changes in hepatic mitochondrial respiratory chain-related gene expression and mitochondrial function-related markers, which may contribute to altered hepatic energy metabolism and fat mobilization. These findings suggest that, unlike orlistat, di-SQ may exert broader metabolic effects, with a more evident impact on alleviating hepatic steatosis than on systemic TG levels or eWAT mass.

Compared with the liver, eWAT appeared less responsive to di-SQ, although microarray analysis still suggested transcript-level changes in eWAT. Previous studies have shown that hepatic LD dynamics can be modulated to improve fatty liver-related features without necessarily altering WAT mass or systemic triglyceride levels (Cho et al., 2023[14]; Boutagy et al., 2024[7]). In addition, LD-associated proteins such as Plin1 and Plin2 may contribute to obesity and NAFLD (Zhang et al., 2024[70]), consistent with our finding that di-SQ altered their expression in the liver and eWAT and promoted the formation of smaller LDs. Together, these findings suggest that di-SQ may primarily influence hepatic lipid metabolism and also contribute to improvements in NAFLD-related features.

In the liver, *Pparg* is normally expressed at low levels but can be induced under conditions such as fatty liver and insulin resistance. In adipocytes, *Pparg* promotes preadipocyte differentiation and enhances fatty acid synthesis and storage, thereby reducing the lipotoxic effects of free fatty acids on other tissues such as muscle and liver (Tontonoz and Spiegelman, 2008[63]). In hepatic metabolism, *Cebpa* regulates gluconeogenesis and lipoprotein synthesis. Although it helps maintain hepatic metabolic function, its expression may become abnormally elevated under insulin-resistant conditions, thereby affecting glucose and fatty acid metabolism. In adipocytes, *Cebpa* acts synergistically with *Pparg* to promote adipocyte maturation and function (Rosen et al., 2002[54]). *Fasn* is a key enzyme in de novo fatty acid synthesis and is important for energy storage and function in both adipocytes and hepatocytes (Raab et al., 2021[49])*. *In our study, the decreased hepatic expression of *Pparg* after di-SQ treatment may be associated with reduced lipogenic activity, contributing to the alleviation of hepatic fat deposition, and may also be consistent with improved insulin-related gene expression. In contrast, hepatic *Fasn* expression did not change significantly, suggesting that di-SQ did not markedly affect hepatic fatty acid synthesis.

In addition, *Cebpa* expression in the liver also did not change significantly, which may indicate that the effect of di-SQ on hepatic adipogenic/lipogenic gene expression was more evident for *Pparg* than for *Cebpa*. Similarly, the significant reduction in *Cebpa* expression in eWAT suggests a suppression of adipogenic activity, which may have contributed to the slight reduction in eWAT mass observed in this study. These findings may reflect a coordinated adjustment in adipocyte metabolic status, characterized by reduced adipogenic drive together with the maintenance of fatty acid synthesis-related expression to meet metabolic demands. In addition, these tissue-specific differences may also involve other regulatory pathways, such as *Nrf2*, Wnt pathway, and thyroid hormone-related signaling.

In glucose metabolism and insulin signaling, AMPK is a key regulator of energy balance and has been associated with insulin sensitivity, mTORC activity (Laplante and Sabatini, 2012[37]), as well as *Foxo3 *regulation (Greer et al., 2007[20]). However, the regulation of FOXO proteins and mTORC may be more influenced by factors such as nutritional status, growth factor levels, and other upstream and downstream signals, including but not limited to AMPK (Brown and Webb, 2018[8]). In our study, di-SQ was associated with altered *Prkaa1* expression, whereas no corresponding normalization was observed in mTOR- and FOXO-related genes. This pattern may reflect contribution from other metabolic pathways, such as PI3K-Akt signaling, which can regulate mTOR (Sarbassov et al., 2005[57]) and *Foxo3* (Greer et al., 2007[20]) independently of AMPK. Similarly, the observed normalization of certain gluconeogenesis-related genes may involve compensatory input from other signaling pathways, including AMPK or GSK-3 (Wang et al., 2022[66]). However, these hypotheses require further validation.

Insulin inhibits the production and release of glucose by the liver by blocking gluconeogenesis and glycogenolysis (Saltiel and Kahn, 2001[55]). This is achieved through both the direct action of insulin on the liver (Michael et al., 2000[42]) and its indirect effects on substrate availability (Bergman and Ader, 2000[6]). New data suggested that the FOXO family of transcription factors may act through phosphorylation of Akt-related protein kinases and the *Pparg* coactivator *Ppargc1* (Yoon et al., 2001[67]). However, our data suggests that the changes in *Pparg* and *Ppargc1a *were more likely associated with AMPK-related regulation than with direct evidence of Akt pathway activation. The deletion of insulin receptors and *Irs1* has been linked to insulin resistance and type 2 diabetes (Brüning et al., 1997[9]), and adipose tissue-specific knockout of *Slc2a4* exacerbates glucose intolerance (Abel et al., 2001[1]). The upregulation of hepatic *Irs1 *and adipose* Slc2a4 *by di-SQ may indicate gene expression patterns potentially associated with improved insulin signaling. Leptin, produced by adipocytes, is a key regulator of energy balance and insulin sensitivity (Picó et al., 2022[46]). Leptin is involved in and influences many signaling pathways. In addition to those mentioned in the main text, the Leptin-STAT3 pathway, Leptin-MAPK/ERK pathway, and their downstream factors are also crucial for fatty acid metabolism, appetite regulation, and insulin resistance. Given that di-SQ affects adipocyte differentiation and lipid storage, it may also influence leptin signaling. This could further contribute to the regulation of insulin signaling in metabolic disorders, although additional functional validation is required. Regarding the smaller size of adipocytes, as we mentioned before, *Cebpa*, a key regulator of adipocyte differentiation and lipid storage, was also significantly inhibited. This inhibition reduced both adipocyte differentiation and lipid storage, resulting in smaller adipocytes.

The mitochondrial respiratory chain in the liver is a critical component of energy production and essential for maintaining cellular metabolic balance and overall health; through oxidative phosphorylation, hepatic mitochondria efficiently generate ATP, the primary energy currency for cells. In metabolic syndrome, mitochondrial function is often impaired, leading to reduced energy production efficiency (Wallace, 2005[65]). When the mitochondrial respiratory chain function is normalized, it can restore normal ATP production and improve both cellular and systemic energy metabolism, which is crucial for reducing insulin resistance and improving glucose homeostasis (Lowell and Shulman, 2005[41]). Abnormalities in the mitochondrial respiratory chain are typically associated with excessive generation of reactive oxygen species (ROS), which can increase oxidative stress and damage cellular structures and functions (Zorov et al., 2014[71]). Mitochondrial function is normalized in patients with metabolic syndrome. ROS production can be reduced, thereby decreasing oxidative stress and contributing to reduced inflammation and improved overall cellular health. Our experimental results showed that di-SQ was associated with partial normalization of hepatic respiratory chain gene expression and increased expression of *Cpt1a* and *Ppargc1a*, along with improved hepatocellular integrity and reduced inflammation, which may reflect improved respiratory chain activity and mitochondrial function.

Although the effects were less pronounced than in the liver, we also examined eWAT to gain a broader understanding of di-SQ's metabolic role, given the lack of prior studies in this tissue. The expression and activity of*f1 Prdm16* and *Ucp1* in eWAT are crucial areas of study in metabolic biology. These two factors play a central role in promoting WAT browning (Bartelt and Heeren, 2014[4]). This browning process enhances the metabolic activity of adipose tissue, improves insulin sensitivity, and helps regulate body weight and blood glucose levels. *Prdm16* is a key regulatory factor in brown adipocyte differentiation. It activates a series of genes involved in brown fat differentiation, including *Ucp1 *and *Ppargc1a* (Kajimura et al., 2008[31]). *Prdm16* increases energy expenditure, contributing to heat production, and weight control, and can also inhibit adipocyte hypertrophy and reduce inflammatory responses (Jiang et al., 2022[30]). *Ucp1* uncouples the mitochondrial respiratory chain from ATP synthesis, releasing energy as heat rather than storing it as ATP (Feldmann et al., 2009[18]). In this study, di-SQ treatment was associated with increased expression of *Prdm16* and *Ucp1 *in eWAT, lending partial support to our hypothesis that di-SQ may promote a transcriptional shift toward browning-related gene programs and mitochondrial metabolism.

There is a close association between obesity-related dysfunctions and hepatic steatosis, particularly in the development and progression of NAFLD. Increased hepatic lipid synthesis, caused by the inability to regulate glucose and lipid metabolism despite normal insulin levels, coupled with a reduced capacity of insulin to inhibit lipolysis, leads to an influx of FFAs into the liver and decreased fatty acid oxidation. This results in an accumulation of LDs within hepatocytes, leading to steatosis (Diehl and Day, 2017[16]). Increased adiponectin levels can activate SIRT1, which in turn promotes fatty acid oxidation and reduces lipotoxicity, potentially counteracting some of the negative effects of hepatic steatosis. As steatosis worsens, cellular stress responses are triggered, activating inflammatory pathways in the liver and leading to the release of inflammatory factors (Tilg and Moschen, 2008[61]), such as *TNF-α* and *IL-6*. Under prolonged inflammatory stimulation, hepatic stellate cells are activated in an attempt to repair liver damage (Friedman, 2003[19]), but excessive fibrosis can lead to liver dysfunction and potentially progress to irreversible cirrhosis (Powell et al., 2021[48]). In our study, di-SQ treatment was associated with reduced hepatic steatosis, lower expression of inflammatory markers, and decreased fibrosis-related gene expression. Given that NAFLD causes an increase in aminotransferase levels (Rinella et al., 2023[52]), and di-SQ treatment significantly reversed hepatic ALT and AST levels, these findings are consistent with an attenuation of NAFLD-associated liver injury, although further validation in dedicated NAFLD models will be required.

WAT, as an important endocrine organ, regulates systemic metabolism by secreting cytokines known as adipokines. In cases of insulin resistance, pro-inflammatory factors interfere with insulin signaling (Hotamisligil, 2006[27]). This reduces glucose uptake and fat storage while increasing FFAs in the bloodstream. These FFAs accumulate in non-adipose tissues, such as the liver and muscles, exacerbating insulin resistance. Additionally, obesity and insulin resistance inhibit the secretion of anti-inflammatory adipokines, such as adiponectin (Gregor and Hotamisligil, 2011[21]; Arita et al., 1999[3]), thereby further triggering WAT inflammation and systemic inflammatory responses. As the main form of energy storage, WAT consists of two types: eWAT and inguinal white adipose tissue (iWAT) (Sanchez-Gurmaches and Guertin, 2014[56]). Specifically, eWAT has lower metabolic activity and accumulates more fat (Pillon et al., 2021[47]) during excessive accumulation, dysfunction, and obesity, playing a crucial role in metabolic diseases such as obesity and diabetes (Zatterale et al., 2020[68]). In contrast, iWAT has higher metabolic activity and is currently considered closely related to the browning/beiging of white adipose tissue (Labbé et al., 2016[36]). Given that our bioinformatics results indicate di-SQ has a relatively weak effect on WAT, we only analyzed eWAT. In eWAT, di-SQ treatment was associated with altered expression of genes related to glucose transport, adipocyte metabolism, and inflammation, suggesting partial normalization of obesity-associated transcriptional dysregulation rather than direct restoration of insulin sensitivity.

As a pioneering study on the metabolic effects of di-SQ, this work provides initial insights, although several limitations should be acknowledged. First, as discussed above, iWAT plays an important role in browning and energy expenditure, while brown and beige adipose tissues are key contributors to thermogenesis. Therefore, the lack of a broader analysis across different adipose depots limits a more complete understanding of the metabolic effects of di-SQ. In addition, serum TG was assessed as a lipid-related marker, and ALT and AST were measured as indicators of liver injury, providing a preliminary evaluation of the metabolic effects of di-SQ. However, other important serum markers, such as cholesterol, LDL, HDL, and additional lipid-related parameters, were not evaluated, which limited a more comprehensive interpretation of its systemic metabolic effects. Furthermore, this study did not include direct assessments of cellular respiration, ROS-related oxidative stress, or peroxisomal lipid metabolism, all of which would help to further clarify the mitochondrial and metabolic mechanisms underlying the effects of di-SQ.

## Conclusion

In this study, di-SQ was associated with changes in hepatic energy metabolism-, gluconeogenesis-, and inflammation-related gene expression, potentially in association with *Sirt1* and/or *Prkaa1*, and with reduced hepatic inflammatory and oxidative stress-related markers. In eWAT, di-SQ was associated with increased *Slc2a4* expression and coordinated changes in genes related to insulin signaling, lipid metabolism, and energy expenditure.

Overall, these findings provide exploratory evidence that di-SQ may have potential to improve obesity-related metabolic dysregulation.

## Declaration

### CRediT authorship contribution statement

**Yu Cheng: **Conceptualization, Investigation, Methodology, Formal analysis, Software, Validation, Visualization, Writing - Original draft; **Munkhzul Ganbold:** Investigation, Methodology, Writing - Review and editing; **Elda Nurafnie Binti Ibnu Rasid:** Investigation, Methodology; **Farhana Ferdousi:** Software, Methodology, Writing - Review and editing; **Hiroko Isoda:** Conceptualization, Supervision, Project administration, Resources, Funding acquisition.

### Funding

This work was supported by Japan Science and Technology (JST), Grant Number JPMJPF2017. The funder had no role in study design; in the collection, analysis and interpretation of data; in the writing of the report; and in the decision to submit the article for publication.

### Conflict of interest

The authors declare no conflict of interest.

### Artificial Intelligence (AI) - assisted technology

Generative artificial intelligence tools were used solely for language refinement and improving the clarity of the manuscript. All scientific content, analyses, interpretations, and conclusions were reviewed and verified by the authors.

### Data availability

The supporting data of this article can be found within this paper and in supplementary files. The microarray data have been deposited in the NCBI GEO database and are publicly available (Accession number: GSE281432).

### Acknowledgments

Yu Cheng received Support for Pioneering Research Initiated by The Next Generation, SPRING scholarship.

## Supplementary Material

Supplementary information

## Figures and Tables

**Figure 1:**
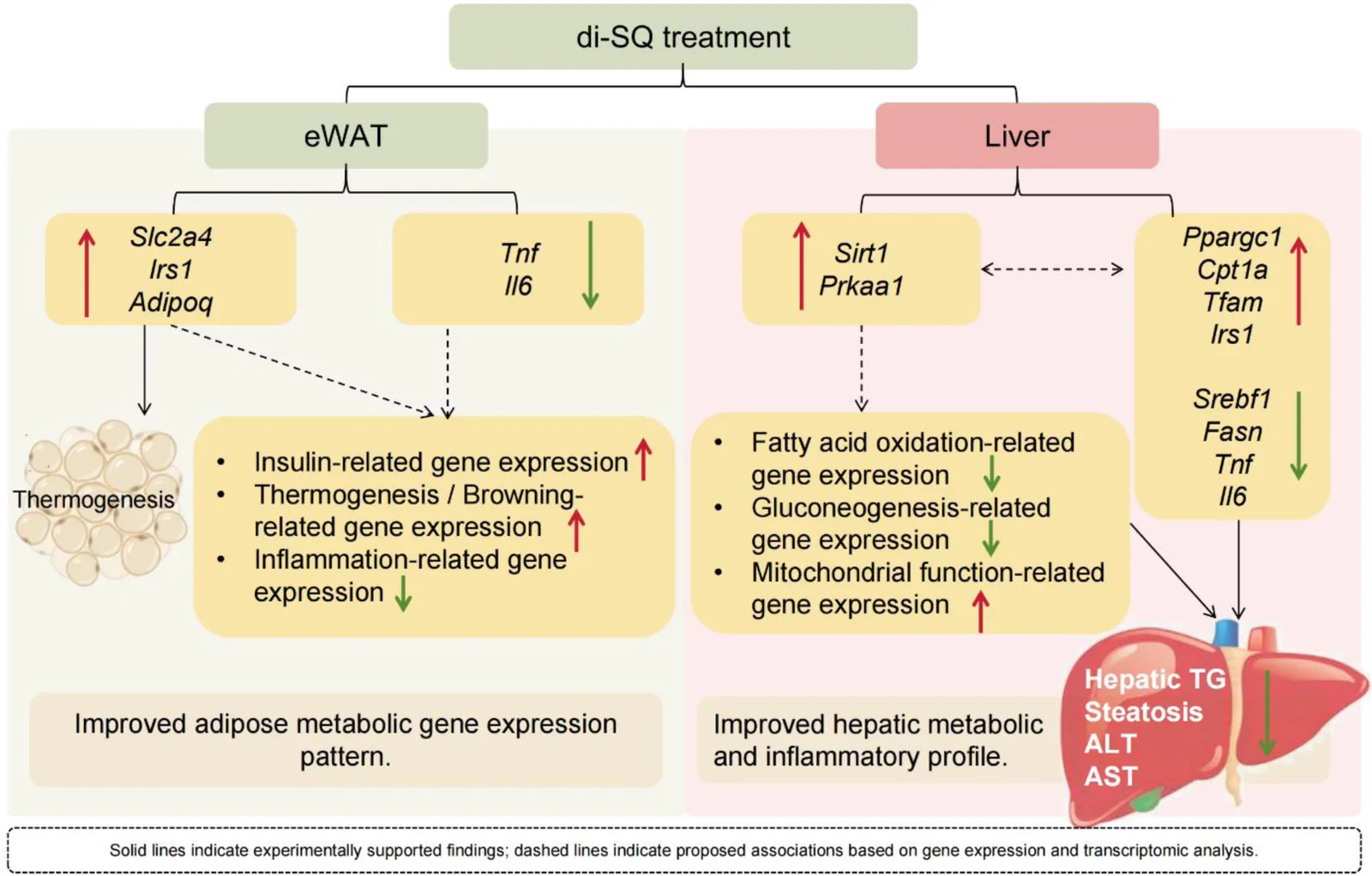
Graphical abstract
