# Supplementary material for: A diethylene glycol oligomer squalene derivative modulates metabolic gene expression in ob/ob mice
Source: EXCLI J. 2026 Sep 7;25:1460–85. doi: 10.17179/excli2026-9373 (PMC13595869; doi:10.17179/excli2026-9373)
Supplement: Supplementary file 1 [file EXCLI-25-1460-s-001.pdf]

EXCLI Journal 2026;25:1460-1485 – ISSN 1611-2156
Received: February 26, 2026, accepted: June 29, 2026, published: September 07, 2026

Supplementary information to:

Original article:

A DIETHYLENE GLYCOL OLIGOMER SQUALENE DERIVATIVE MODULATES METABOLIC GENE EXPRESSION IN OB/OB MICE

Yu Cheng[1], Munkhzul Ganbold[2], Elda Nurafnie Binti Ibnu Rasid[1], Farhana Ferdousi[1,3,4], Hiroko Isoda[1,2,3,4*]

1 Tsukuba Life Science Innovation (T-LSI) Program, Graduate School of Science and Technology, University of Tsukuba, Tsukuba 305-8577, Japan
2 Open Innovation Laboratory for Food and Medicinal Resource Engineering (FoodMed-OIL), National Institute of Advanced Industrial Science and Technology (AIST), Tsukuba, Ibaraki 305-0821, Japan
3 Institute of Life and Environmental Sciences, University of Tsukuba, Tsukuba 305-8577, Japan
4 Alliance for Research on the Mediterranean and North Africa (ARENA), University of Tsukuba, Tsukuba 305-8577, Japan

* **Corresponding author:** Professor Hiroko Isoda, Institute of Life and Environmental Sciences, University of Tsukuba, Cooperative Research Building A302, 1-1-1 Tennodai, Tsukuba, Ibaraki 305-8572, Japan; Tel: 029-853-3982; Fax: 029-853-5776; E-mail: isoda.hiroko.ga@u.tsukuba.ac.jp

https://dx.doi.org/10.17179/excli2026-9373

This is an Open Access article distributed under the terms of the Creative Commons Attribution License (https://creativecommons.org/licenses/by/4.0/).

Supplementary Method 1: Online bioinformatics analysis tools and access dates

1. Database for Annotation, Visualization and Integrated Discovery (DAVID, 2021) online bioinformatics tools (https://david.ncifcrf.gov/; Accessed on Dec. 20th, 2023)
2. SRplot online tool (https://www.bioinformatics.com.cn/srplot; Accessed on Dec. 26th, 2023 – Nov. 29th, 2024)
3. ExpressAnalyst online platform (https://www.expressanalyst.ca/; Accessed on Nov. 19th, 2024 – Nov. 25th, 2024)
4. Protein-protein interaction (PPI) networks were created using the NetworkAnalyst online platform (https://www.networkanalyst.ca/NetworkAnalyst/home.xhtml; Accessed on Nov. 19th, 2024 – Dec. 8th, 2024)
5. Elabscience, Inc. (https://www.elabscience.com)

EXCLI Journal 2026;25:1460-1485 – ISSN 1611-2156
Received: February 26, 2026, accepted: June 29, 2026, published: September 07, 2026

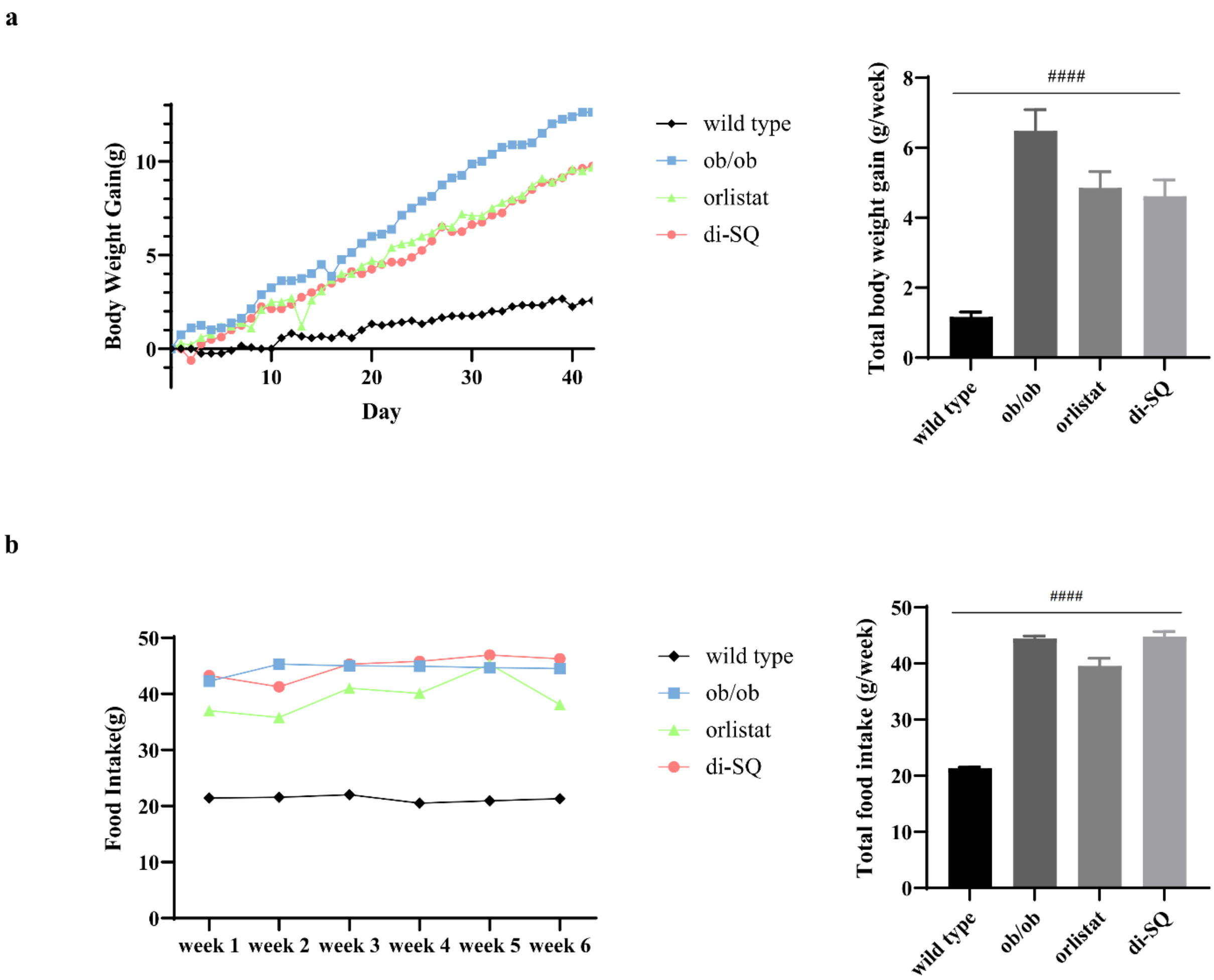

Supplementary Figure 1: Effects of di-SQ treatment on body weight and food intake in ob/ob mice and wild-type mice. (**a**) Body weight changes (left) and total body weight gain (right) of both wild type mice and ob/ob mice. (**b**) Food intake (left) and average food intake per week (right) of both wild type mice and ob/ob mice during the experimental period. n = 5 mice per group.

EXCLI Journal 2026;25:1460-1485 – ISSN 1611-2156
Received: February 26, 2026, accepted: June 29, 2026, published: September 07, 2026

ob/ob

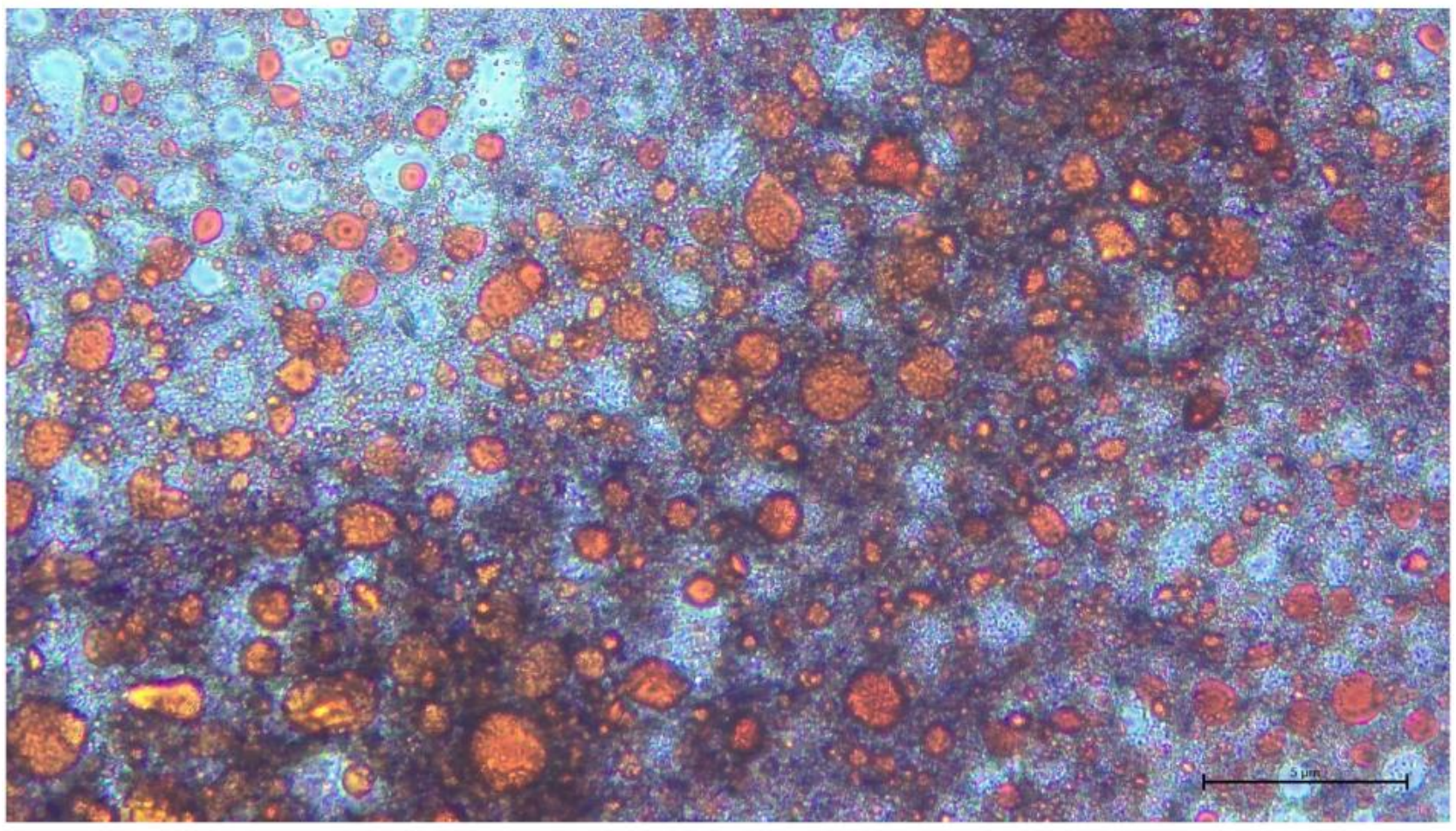

Supplementary fig 2a. Oil red O staining of liver. Scale bar = 5 μm.

orlistat

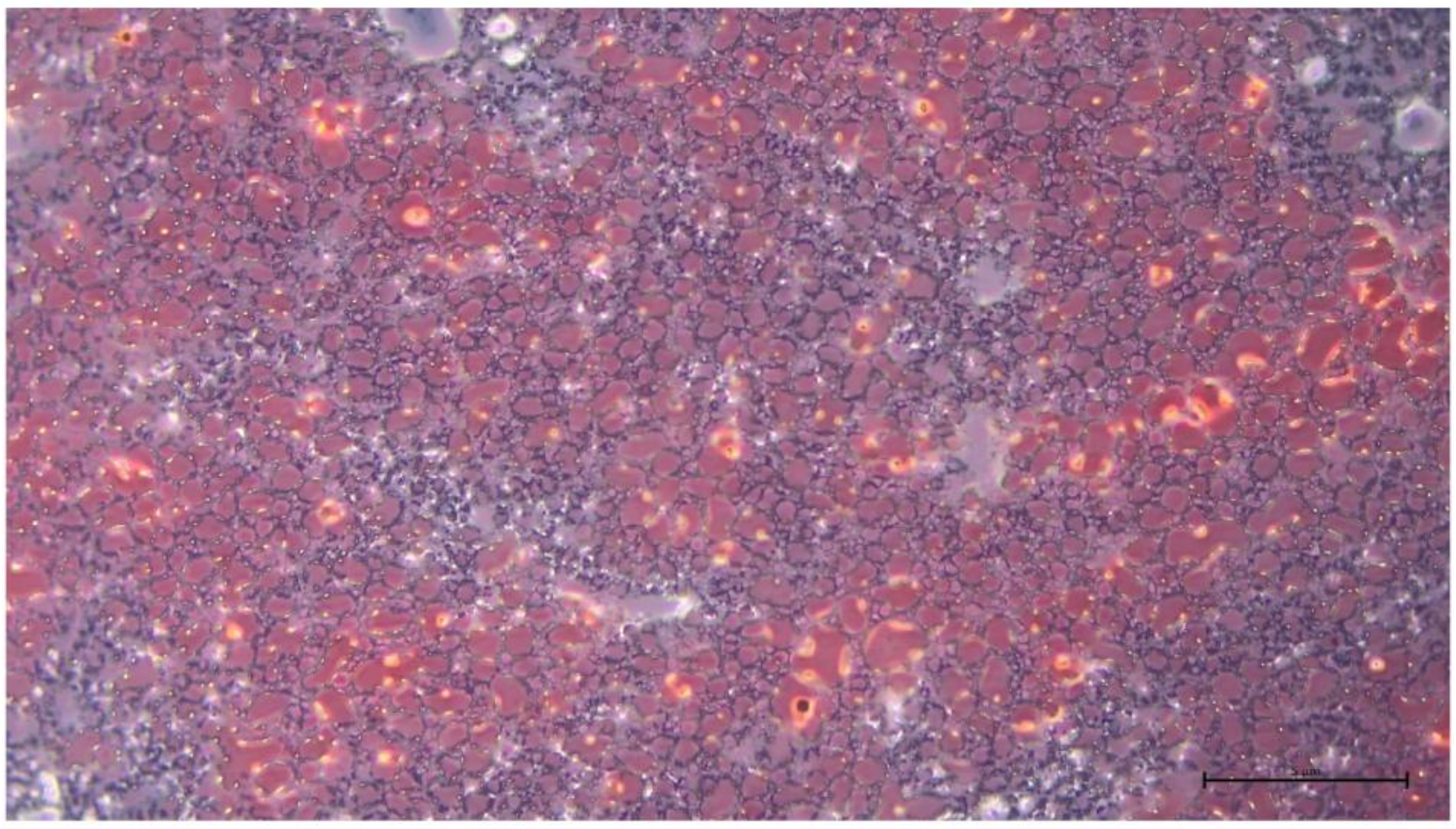

Supplementary fig 2b. Oil red O staining of liver. Scale bar = 5 μm.

di-SQ

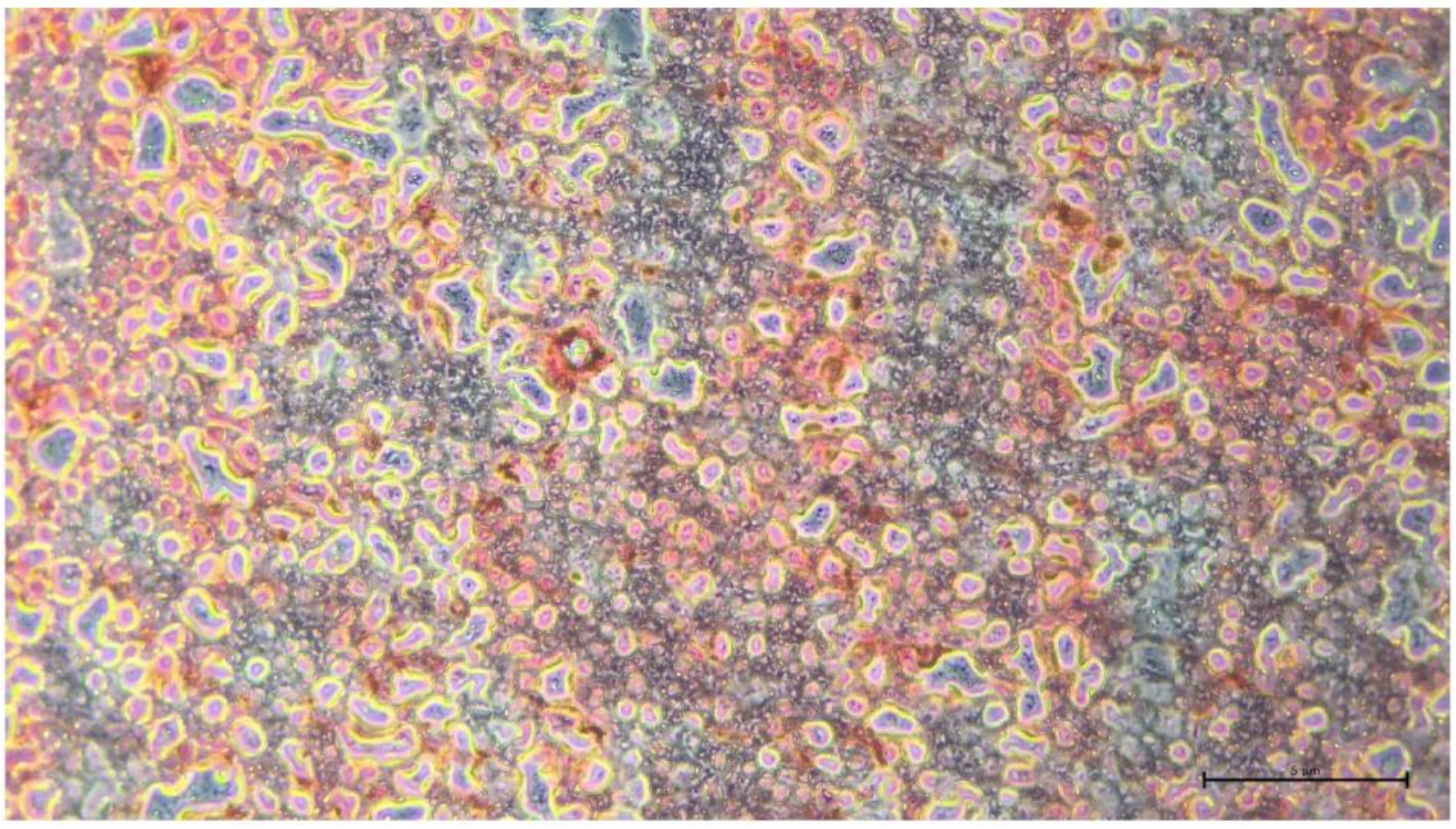

Supplementary fig 2c. Oil red O staining of liver. Scale bar = 5 μm.

Supplementary Figure 2: Original images of oil red O staining. (**a**) ob/ob mice, (**b**) orlistat treated mice, (**c**) di-SQ treated mice.

EXCLI Journal 2026;25:1460-1485 – ISSN 1611-2156
Received: February 26, 2026, accepted: June 29, 2026, published: September 07, 2026

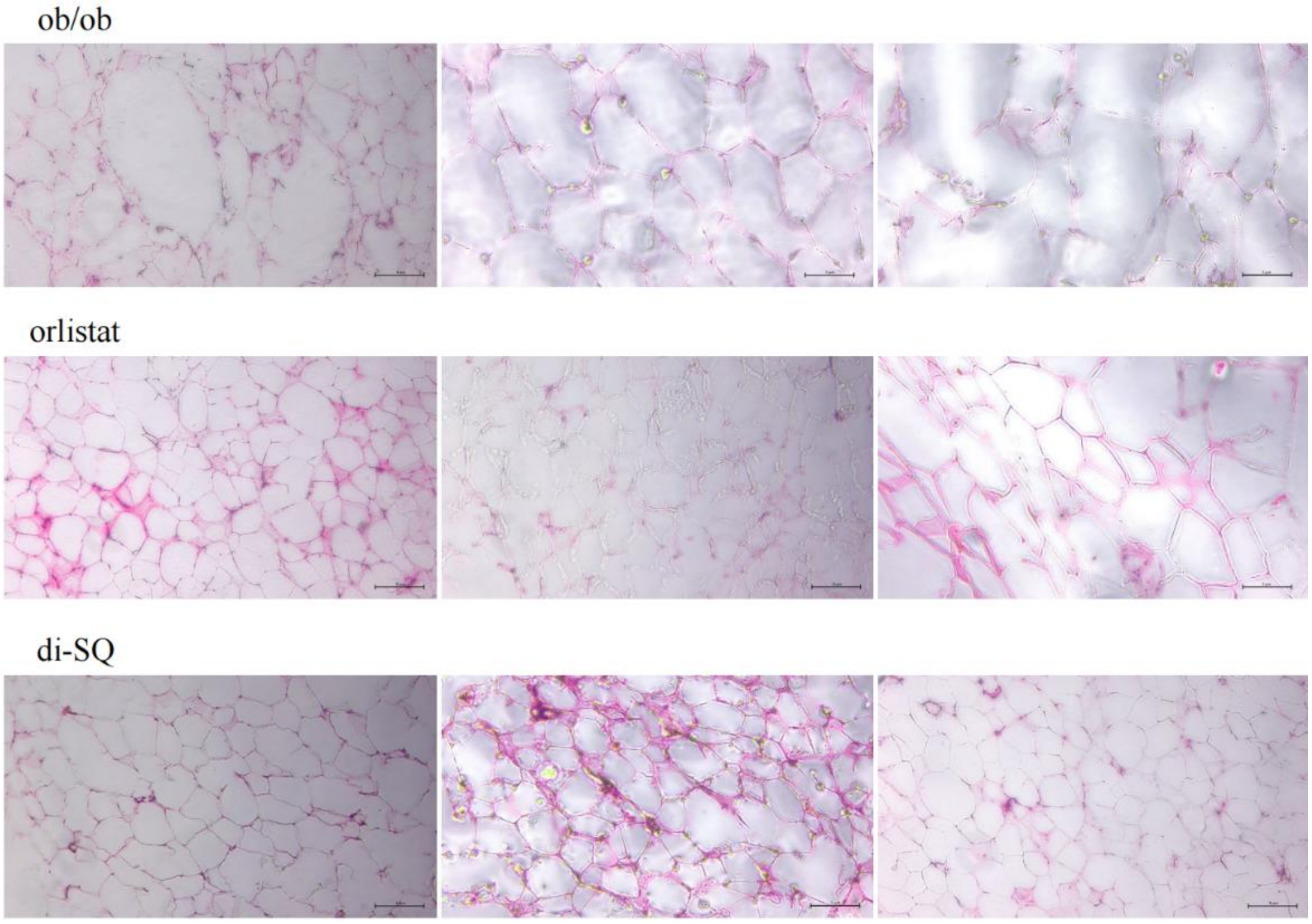

Supplementary Figure 3: Representative histological images of eWAT from each treatment group (n = 3 per group). These images served as the basis for manual adipocyte area quantification using ImageJ. Scale bar = 5 µm.

Supplementary Table 1: Effects of di-SQ treatment on gene expressions related to metabolism-related diseases in the liver

Liver	Gene Symbol	ob/ob	wild type	di-SQ
**Adipogenesis/ Lipogenesis**	*Adipoq*	3.37 ± 0.13	4.13 ± 0.08 **	3.94 ± 0.1 *
	Cebpa	13.51 ± 0.2	11.98 ± 0.47 ***	13.28 ± 0.18
	Pparg	11.21 ± 0.16	6.76 ± 0.4 ***	10.62 ± 0.25
	Srebf1	10.36 ± 0.09	9.43 ± 1.45	9.62 ± 0.28
	Fasn	15.18 ± 0.2	12.29 ± 0.46 ***	15.6 ± 0.11
**Lipid droplet size**	*Plin1*	4.86 ± 0.2	3.73 ± 0.2 ***	4.24 ± 0.09 **
	Plin2	16.09 ± 0.1	13.09 ± 0.24 ***	15.51 ± 0.08
	Lpin1	11.28 ± 0.53	14.37 ± 0.25 ***	12.99 ± 0.25 ***
**NASH related**	*Col1a1*	7.1 ± 0.47	6.56 ± 0.49	6.76 ± 0.21
	Tgfβ1	8.63 ± 0.36	7.57 ± 0.5 *	8.21 ± 0.12 *
**Mitochondrial function**	*Nrf1*	8.13 ± 0.14	8.4 ± 0.35	8.31 ± 0.37
	Pgc1a	6.93 ± 1.4	8.75 ± 1.06	9.01 ± 0.16 *
	Tfam	8.57 ± 0.46	9.63 ± 0.56 *	10 ± 0.19 *
	Cpt1a	12.3 ± 0.38	13.55 ± 0.85 *	13.85 ± 0.15 *
**Hepatic insulin signaling**	*Igf1r*	5.3 ± 0.03	5.9 ± 0.39 *	5.63 ± 0.12
	Gck	9.89 ± 0.65	10.94 ± 0.27	10.6 ± 0.15
**Hepatic inflammation**	*Il6*	4.46 ± 0.52	4.19 ± 0.34	3.9 ± 0.29 *
	Tnf	3.69 ± 0.4	3.1 ± 0.13	3.25 ± 0.2

The data are from the signal intensity of the microarray experiment, and the average of three sets of repeated data is taken. Values are expressed as mean ± SEM. The mean and SEM were calculated using Microsoft Excel, with the mean obtained through the AVERAGE function and SEM calculated as STDEV.S/√n. Statistical significance: *p < 0.05, **p < 0.01, ***p < 0.001, ****p < 0. 0001.

EXCLI Journal 2026;25:1460-1485 – ISSN 1611-2156
Received: February 26, 2026, accepted: June 29, 2026, published: September 07, 2026

Supplementary Table 2: Effects of di-SQ treatment on gene expressions related to metabolism-related diseases in eWAT

eWAT	Gene Symbol	ob/ob	wild type	di-SQ
Adipogenesis/ Lipogenesis	*Adipoq*	16.32 ± 1.44	18.27 ± 0.36 *	18.33 ± 0.18 *
	Cebpa	13.64 ± 0.48	12.51 ± 0.43 **	12.14 ± 2.22
	Pparg	14.23 ± 0.45	13.23 ± 0.39	12.87 ± 2.44
	Srebf1	7.67 ± 0.11	7.22 ± 0.46	7.48 ± 0.35
	Fasn	15.75 ± 0.31	14.07 ± 0.81	13.56 ± 0.36 *
Lipid droplet size	*Plin1*	14.84 ± 0.15	13.66 ± 0.26 **	13.79 ± 0.3 **
	Plin2	16.4 ± 0.25	10.57 ± 1.1 **	14.55 ± 2.26 *
	Lpin1	14.18 ± 0.25	14.68 ± 0.28	14.64 ± 0.19
Diabetes related	*Insr*	12.38 ± 0.3	13.51 ± 0.49 **	12.85 ± 0.26 *
	Igf1r	6.29 ± 0.42	8.33 ± 0.6 *	8.1 ± 2.2 *
Mitochondrial function	*Gck*	5.58 ± 0.27	5.8 ± 0.25	5.89 ± 0.67
	Pgc1a	6.34 ± 0.21	8.68 ± 1.45 **	7.06 ± 0.09 *
	Tfam	7.2 ± 0.39	8.1 ± 0.35 *	7.55 ± 0.06
	Sirt3	8.61 ± 0.11	8.79 ± 0.54	8.97 ± 0.53
eWAT thermogenesis	*Prdm16*	4.1 ± 0.01	5.45 ± 0.39	5.91 ± 0.22 *
	Ucp1	3.8 ± 0.06	4.37 ± 0.24	3.83 ± 0.5
Inflammation	*Il6*	4.55 ± 0.15	4.29 ± 0.52	4.28 ± 0.4
	Tnf	5.34 ± 0.25	3.11 ± 0.05 ***	3.65 ± 0.57 ***

The data are from the signal intensity of the microarray experiment, and the average of three sets of repeated data is taken. Values are expressed as mean ± SEM. The mean and SEM were calculated using Microsoft Excel, with the mean obtained through the AVERAGE function and SEM calculated as STDEV.S/√n. Statistical significance: *p < 0.05, **p < 0.01, ***p < 0.001, ****p < 0. 0001.
